# TOPK Suppresses the CD8^+^ T Cell Antitumor Immunity via Modulation of IRF5 Expression

**DOI:** 10.34133/cancomm.0021

**Published:** 2026-03-20

**Authors:** Nianke Zang, Jinfeng Gan, Ye Chen, Zheng Huang, Chichu Xie, Junlong Dang, Changyuan Huang, Linjie Yang, Xuelian Chen, Guangli Rong, Jianbo Sun, Yiming Shao, Julie Wang, Guangying Qi, Yu Liu, Song Guo Zheng

**Affiliations:** ^1^ Clinical Research Center, The First Dongguan Affiliated Hospital, Guangdong Medical University, Dongguan, Guangdong, P. R. China.; ^2^ Guangxi Key Laboratory of Tumor Immunology and Microenvironmental Regulation, Guilin Medical University, Guilin, Guangxi, P. R. China.; ^3^ Guangxi Health Commission Key Laboratory of Tumor Immunology and Receptor-Targeted Drug Basic Research, Guilin Medical University, Guilin, Guangxi, P. R. China.; ^4^ Department of Immunology, School of Cell and Gene Therapy, Songjiang Institute and Songjiang Hospital Affiliated to the Shanghai Jiao Tong University School of Medicine, Shanghai, P. R. China.; ^5^ State Key Laboratory of Pathogenesis, Prevention and Treatment of High Incidence Diseases in Central Asia, The First Dongguan Affiliated Hospital, Guangdong Medical University, Dongguan, Guangdong, P. R. China.; ^6^ Dongguan Key Laboratory of Chronic Inflammatory Diseases, The First Dongguan Affiliated Hospital, Guangdong Medical University, Dongguan, Guangdong, P. R. China.; ^7^ The Key Laboratory of Sepsis Translational Medicine, Guangdong Medical University; Dongguan Key Laboratory of Sepsis Translational Medicine; The First Dongguan Affiliated Hospital, Guangdong Medical University, Dongguan, Guangdong, P. R. China.; ^8^ Guangdong Provincial Key Laboratory of Medical Immunology and Molecular Diagnostics, School of Medical Technology, Guangdong Medical University, Dongguan, Guangdong, P. R. China.; ^9^ State Key Laboratory of Innovative Immunotherapy, Shanghai Jiao Tong University, Shanghai, P. R. China.

## Abstract

**Background:** T-LAK cell-originated protein kinase (TOPK), a serine/threonine kinase, is aberrantly overexpressed in human tumors and promotes malignant proliferation. Melanoma is a highly immunogenic tumor in which CD8^+^ T cell-mediated cytotoxicity is usually less effective in tumor control and responsive to immune checkpoint blockade. It is unclear whether the expression and functional characterization of TOPK within the immune cells affect the tumor microenvironment (TME) in patients with melanoma. This study aims to elucidate the expression pattern and immunoregulatory function of TOPK in CD8^+^ T lymphocytes during antitumor responses. **Methods:** Public single-cell RNA-sequencing (scRNA-seq) dataset analysis and flow cytometry assessed TOPK in tumor-infiltrating CD8^+^ T cells from patients with melanoma. Genetic deletion and pharmacological inhibition of TOPK using HI-TOPK-032 tested T cell-mediated melanoma control. Flow cytometry and tumor cell coculture killing assays measured effector release and target-cell apoptosis. Mechanistic analyses included assessment of interferon regulatory factor 5 (IRF5) expression, together with combination therapy using a programmed cell death protein 1 (PD-1)-blocking antibody in vivo. scRNA-seq of tumor-infiltrating lymphocytes (TILs) from *Topk*^fl/fl^ and *Cd8*^Cre^*Topk*^fl/fl^ mice was also performed to define TOPK-dependent immune programs within the melanoma TME. **Results:** Single-cell transcriptomes identified a TOPK^+^ subset of tumor-infiltrating CD8^+^ T cells in melanoma, which was higher than that in normal lymph nodes (LNs), and exhibited suppressed cytotoxic and cytokine programs. CD8^+^ T cell-specific *Topk* deletion increased granzyme B (GzmB), tumor necrosis factor-α (TNF-α), and interferon-γ (IFN-γ) secretion and improved tumor control. TOPK-deficient CD8^+^ T cells showed elevated activation-associated signaling pathways and immune effector gene expression. In murine TIL scRNA-seq, *Cd8*^Cre^*Topk*^fl/fl^ tumors exhibited increased effector and activation programs, reduced exhaustion and dysfunction programs, and enhanced immune crosstalk in the TME. Mechanistically, TOPK suppressed IRF5 expression and HI-TOPK-032 restored CD8^+^ T cell cytotoxicity in vitro and, with anti-PD-1, further inhibited tumor growth and increased intratumoral cytokine production. In human CD8^+^ T cells, enforced TOPK expression impaired cytotoxicity and cytokine secretion, reversed by IRF5 coexpression. **Conclusions:** These findings establish TOPK as the immune checkpoint limiting CD8^+^ T cell functionality in tumors and indicate the potential of TOPK inhibition as a strategy to augment T cell-based immunotherapies.

## Background

Melanoma persists as one of the most formidable cutaneous malignancies in clinical management, primarily because of its high potential for metastasis, development of treatment resistance, and tumor heterogeneity [1–3]. Although patient survival has been substantially improved with targeted therapies, such as B-RAF proto-oncogene serine/threonine kinase (BRAF) [4,5]/mitogen-activated protein kinase kinase (MEK) inhibitors [6], and immune checkpoint blockade (ICB) therapies, including anti-programmed cell death protein 1 (anti-PD-1) [7,8] and anti-cytotoxic T-lymphocyte-associated antigen 4 (anti-CTLA-4) antibodies [9,10], long-term outcomes remain suboptimal. In the pivotal KEYNOTE-006 trial, the 5-year overall survival (OS) of patients with pembrolizumab monotherapy was 38.7% [11], while an extended follow-up study (KEYNOTE-587) reported a 5-year OS of 92.9% and a 5-year progression-free survival (PFS) of 70.1% among patients who completed ≥94 weeks of therapy [12]. Similarly, the CheckMate-067 study reported a 5-year OS of 52% with nivolumab plus ipilimumab, and in patients with resected stage IIIb/IIIc/IV melanoma, the 5-year OS rates reached 76% with nivolumab and 72% with ipilimumab [13,14]. Despite these advances, approximately 60% of patients with advanced-stage disease still develop primary or acquired resistance [15], highlighting the persistent therapeutic challenge. The crux of this treatment challenge lies within the complex immunosuppressive network of the tumor microenvironment (TME), where CD8^+^ T cell exhaustion—a state of progressive functional impairment—serves as a key driver of immune escape [16–18]. This exhaustion process​ involves a gradual loss of effector functions [e.g., reduced production of interferon-γ (IFN-γ) and tumor necrosis factor-α (TNF-α)] under persistent antigen exposure [19,20], ​epigenetic reprogramming, and co-up-regulation of inhibitory receptors [e.g., PD-1 and lymphocyte activation gene 3 (LAG-3)] [21,22]. Consequently, elucidating the molecular drivers of ​T cell exhaustion​ and developing targeted reprogramming strategies are critical steps for overcoming the current limitations in melanoma immunotherapy.

The efficacy of ICB therapies has definitively established the critical role of T cell-mediated antitumor immunity in modern oncology [23,24]. As primary executors of tumoricidal activity, CD8^+^ T cells eliminate malignant cells through 2 principal mechanisms: (a) direct cytolysis via perforin/granzyme B (GzmB) secretion and (b) immunomodulatory signaling through effector cytokines (e.g., IFN-γ and TNF-α), while concurrently establishing antigen-specific immunological memory [22,25]. Nevertheless, during melanoma progression, sustained antigenic stimulation coupled with immunosuppressive cytokines—such as transforming growth factor-β (TGF-β), interleukin-10 (IL-10), and adenosine—within the TME induces progressive transition of CD8^+^ T cells into an exhausted state. These cytokines also promote regulatory T cell (Treg) differentiation and production [26–28], additionally contributing to immune suppression against tumors. This pathophysiological transformation is characterized by 4 hallmarks: (a) functional collapse marked by diminished effector molecule production [25,29], (b) coordinated up-regulation of multiple inhibitory checkpoints (e.g., PD-1 and LAG-3) [30–32] and T cell immunoreceptor with Ig and ITIM domains (TIGIT) [33,34], (c) terminal differentiation arrest mediated by epigenetic reprogramming, and (d) reduced proliferation capacity [35,36].

T-LAK cell-originated protein kinase (TOPK), a key kinase in the mitogen-activated protein kinase (MAPK) signaling pathway, demonstrates aberrant overexpression in solid tumors, including melanoma, where it promotes tumor proliferation and metastasis through the activation of extracellular signal-regulated kinase (ERK) and signal transducer and activator of transcription 3 (STAT3) [37–41]. Previous studies have shown that TOPK promotes tumor growth across multiple cancers and that its inhibition suppresses tumor progression and enhances antitumor immunity—for instance, by improving the efficacy of chimeric antigen receptor (CAR)-T cell in hepatocellular carcinoma and increasing CD8^+^ T cell infiltration and function in renal cell carcinoma [42–44]. However, the immunological functions of TOPK in immune cells, particularly CD8^+^ T lymphocytes, remain poorly characterized, with its role in antitumor immunity remaining completely unexplored.

In this study, we investigated the expression and immunomodulatory role of TOPK in CD8^+^ T cells within the melanoma TME. We analyzed single-cell transcriptomic data to determine whether TOPK expression in tumor-infiltrating CD8^+^ T cells differed from that in normal lymph nodes (LNs). We further examined its association with pathological tumor grades and explored its regulatory relationship with interferon regulatory factor 5 (IRF5), a transcription factor essential for T cell receptor (TCR)-initiated signaling and cytokine gene transcription [45]. The overarching goal of this study was to elucidate the potential role of the TOPK–IRF5 axis in modulating CD8^+^ T cell function and to provide mechanistic insights into T cell-mediated antitumor immunity in melanoma.

## Materials and Methods

### Mice

C57BL/6 mice were purchased from Slac Jingda Experimental Animal Co. Ltd. *TOPK* global knockout (*Topk*^−/−^) mice were kindly provided by F. Zhu (Medical and Industry Crossover Research Institute of Medical College, Henan University), while *Cd8*^Cre^*Topk*^fl/fl^ mice were acquired from Cyagen Biosciences Inc. Mice used in the experiments were aged 6 to 12 weeks old. Both male and female mice were used as the model has no gender difference. All mice were housed in individually ventilated cages (IVCs) within climate-controlled animal facilities maintained under a 14-h light/10-h dark cycle, with ambient temperature regulated at 26 °C, relative humidity at 30% to 70%, and 10 to 15 air changes per hour. The animal experiments were conducted in the specific pathogen-free (SPF) animal facility at the Animal Center of Guangdong Medical University, after obtaining ethical approval from the center (GDY2402030). CRISPR/Cas9-mediated knockout and Cre-loxP-based conditional knockout mouse models were genotyped and validated following the standard molecular and breeding protocols.

### Human samples

Frozen CD8^+^ T cells from healthy donor were obtained from ORIBIOTECH Co. Ltd. Melanoma tumor tissue sections from 90 patients were commercially obtained from Yaxiang Biotechnology Co. Ltd. All human peripheral blood samples and tumor tissue sections used in this study were obtained with ethical approval (approval nos. 202409 and KW250618) and informed consent, as detailed in the Ethical Approval section.

### Cell lines

The murine melanoma cell line B16-F10, the murine colon adenocarcinoma cell line MC38, and the human melanoma cell line A375 were obtained from the Cell Bank of the China Academy of Sciences and cultured in Dulbecco’s modified Eagle’s medium (DMEM; C11995500BT, Gibco) supplemented with 10% fetal bovine serum (FBS; FSP500, Excell) and 1% penicillin–streptomycin (Pen–Strep; 15140148, Gibco), under standard conditions (37 °C, 5% CO₂). B16-F10, MC38, and A375 cell lines were authenticated by short tandem repeat (STR) profiling upon receipt and within 6 months prior to key experiments.

The human Jurkat T cell line (clone E6-1) with TOPK knockout (TOPK-KO) was generated by Cyagen Biosciences Inc. using CRISPR/Cas9-mediated genome editing to introduce a 5-base pair insertion in the *TOPK* coding region, followed by single-cell cloning. Cells were maintained in RPMI 1640 supplemented with 10% FBS and 1% Pen–Strep under standard culture conditions (37 °C, 5% CO₂). TOPK-KO was validated by Sanger sequencing of the edited locus upon receipt.

### Multiplex immunofluorescence staining on paraffin sections

Tissue microarrays of patient samples (YP-MME981) were purchased from Yaxiang Biotechnology Co. Ltd. The slides were sequentially dewaxed using xylene (X112051, Aladdin) and rehydrated through an ethanol (E111962, Aladdin) gradient. The paraffin-embedded tissue sections were deparaffinized and rehydrated, followed by heat-induced antigen retrieval in EDTA retrieval buffer (C1034, Solarbio) using a microwave (boiling, then maintained at sub-boiling temperature) for 15 to 20 min, and allowed to cool to room temperature. Subsequently, to block endogenous peroxidase activity, the sections were immersed in a 3% H_2_O_2_ solution (P0100A, Beyotime) at 25 °C for 10 min. Then, blocking buffer (5% normal goat serum, C0265, Beyotime) was used to block nonspecific binding sites, followed by incubation for 30 min. The anti-CD8 primary antibody (1:200, ab245118, Abcam) was added and incubated overnight at 4 °C. After washing, species-specific poly-horseradish peroxidase (HRP) secondary antibodies (YP-IHC024-HRP, Xiabukang) were added and incubated at 25 °C for 10 to 20 min. The target antigens were visualized using tyramide signal amplification fluorescent staining solution (YP-IHC024-TSA, Xiabukang). Following antibody stripping treatment, the primary and secondary antibody incubation steps were repeated for staining TOPK (1:500, ab236872, Abcam). Finally, 4′,6-diamidino-2-phenylindole (DAPI) (C1002, Beyotime) staining was performed for 5 to 20 min to visualize the cell nuclei. After washing with phosphate-buffered saline (PBS; C10010500BT, Gibco), the slides were mounted with an anti-fade mounting medium and scanned using a Pannoramic MIDI system (3DHISTECH). The results were read using CaseViewer (v2.6.0.166179, 3DHISTECH).

### Tissue microarray cell segmentation

Multiplex fluorescence images (DAPI, nuclei; green, CD8; red, TOPK) were processed to segment CD8^+^ T cell regions (diameter parameter auto-optimized based on tissue morphology) using the Cellpose (v2.0) cyto2 model [46]. Masks were manually verified for segmentation accuracy and then applied to the TOPK (red) channel. TOPK fluorescence intensity within CD8^+^ T cell regions was quantified using integrated density measurement with ImageJ (v1.53e, National Institutes of Health), and mean fluorescence intensity (MFI) was calculated by normalization to the mask area. Trends in TOPK MFI of CD8^+^ T cells across ordered melanoma pathological grades were assessed using the Jonckheere–Terpstra trend test. The relevant workflow is shown in Fig. S1.

### Tissue preparation and flow cytometry

Peripheral blood mononuclear cells (PBMCs) were isolated by layering PBS-diluted murine blood over Ficoll solution (07851, Stemcell) and centrifuging at 900*g* for 30 min (acceleration/deceleration: 1/0). Leukocytes were collected, washed with RPMI 1640 (C11875500BT, Gibco), and resuspended. Spleen (SP) and LN tissues (axillary, inguinal, mesenteric) were mechanically dissociated by passing through a 40-μm strainer (CSS013040, BIOFIL), followed by centrifugation (500*g*, 5 min). Splenocytes underwent red blood cell lysis using ACK buffer (1 min, 25 °C, 11814389001, Roche), which was neutralized with RPMI 1640, followed by centrifugation. For tumor-infiltrating lymphocytes (TILs), minced tumor tissue was digested using collagenase IV (1 mg/ml, C4-BIOC, 37 °C, 30 min, Sigma-Aldrich), filtered, and separated using a discontinuous Percoll (17089109, Cytiva) gradient (70%/40%) at 900*g* for 30 min. Leukocytes at the gradient interface were washed and resuspended in RPMI 1640.

For extracellular antigen staining, antibodies (see Table S1 for antibody name, dilution, catalog number, and supplier) was added to the cells and incubated at 4 °C in the dark for 30 min. The cells were then washed with PBS. For intracellular antigen staining, cells were first treated with the eBioscience Intracellular Fixation & Permeabilization Buffer Kit (88-8824-00, Thermo Fisher), followed by the addition of an appropriate number of antibodies, and incubated overnight at 4 °C in the dark. Similarly, for nuclear antigen staining, the cells were treated with the eBioscience Foxp3/Transcription Factor Staining Buffer Set (00-5523-00, Thermo Fisher), followed by the addition of an appropriate number of antibodies, and incubated overnight at 4 °C in the dark.

For cytokine staining, the cells were first stimulated for 4 h in culture medium containing phorbol 12-myristate 13-acetate (50 ng/ml, M4647, AbMole Bioscience), ionomycin calcium salt (1 μM, I3909, Sigma-Aldrich), and brefeldin A solution (420601, BioLegend). After stimulation, surface staining was performed, followed by treatment with the eBioscience Intracellular Fixation *&* Permeabilization Buffer Kit (88-8824-00, Thermo Fisher). The appropriate amounts of antibodies were then added, and the cells were incubated overnight at 4 °C in the dark. For antibodies without fluorescent tag, primary antibodies were first applied, followed by washing with PBS. Fluorescently labeled secondary antibodies were then added, and the cells were incubated at 4 °C in the dark for 30 min. Finally, cells were washed with PBS, resuspended in staining buffer, and analyzed using a flow cytometer (Agilent Novocyte Opteon and Agilent Novocyte D3000). The flow cytometry results were analyzed using FlowJo software (v10.8.1, Tree Star), with protein expression levels described using MFI. The gating strategy used for flow cytometry analysis is shown in Fig. S2.

### Subcutaneous tumor models

B16-F10 melanoma cells (5 × 10^5^ cells per mouse in 100 μl of PBS) and MC38 colorectal cancer cells (5 × 10^5^ cells per mouse in 100 μl of PBS) were subcutaneously injected into the right flank of 6- to 8-week-old C57BL/6 mice. This inoculum was used for routine tumor growth experiments to ensure robust and consistent tumor establishment. The tumor volume was monitored daily using caliper measurements (tumor volume = length × width^2^ × 0.5). Mice were humanely euthanized with isoflurane (R510-22, Ruiwode) anesthesia, followed by cervical dislocation, when the tumor size reached 1,800 mm^3^. Excised tumors were weighed, photographed, and digested using collagenase IV (1 mg/ml, C4-BIOC, 37 °C, 30 min, Sigma-Aldrich) and deoxyribonuclease I (0.15 mg/ml, 18047019, Invitrogen) for 30 min at 37 °C to prepare single-cell suspensions for downstream analysis.

### CD8^+^ T cell adoptive transfer therapy

Subcutaneous melanoma models were established in 6- to 8-week-old C57BL/6 mice by injecting 1 × 10^5^ B16-F10 cells into the right flank. A lower inoculum was used in adoptive transfer experiments to generate a slower-growing tumor and provide an appropriate therapeutic window for subsequent CD8^+^ T cell transfer. Cyclophosphamide (2 mg per mouse, HY17420, MedChemExpress) was administered intraperitoneally on the following day. On day 3 post-inoculation, the LNs and SPs were harvested from wild-type (WT) and *Topk*^−/−^ mice, and lymphocytes were isolated. Single-cell suspensions were labeled with phycoerythrin (PE)-Cyanine7 anti-mouse CD8a antibody (1 μl/sample, 100722, BioLegend) at 4 °C for 30 min, followed by fluorescence-activated cell sorting (FACS) using a Cytek Aurora CS (Fremont) to purify CD8^+^ T cells. Purified cells (1 × 10^6^ cells in 100 μl of PBS per mouse) were adoptively transferred via tail vein injection into tumor-bearing mice. Intratumoral co-injections of CpG 1826 (1 μg per mouse; sequence, 5′-TCCATGACGTTCCTGACGTT-3′; synthesized by Sangon Biotech) and polyinosinic:polycytidylic acid [poly(I:C)] (1 μg per mouse, tlrl-pic, InvivoGen) were administered on days 12, 14, and 16 post-tumor inoculation [47]. Mice were euthanized on day 26, and tumors were excised, photographed, and measured (tumor volume = 0.5 × length × width^2^).

### Quantitative real-time PCR

An equal amount of total RNA extracted from cells using RNAprep Pure Cell/Bacteria Kit (DP430, TIANGEN) was reverse transcribed into cDNA using RevertAid First Strand cDNA Synthesis Kit (K1622, Thermo Fisher). Quantitative real-time polymerase chain reaction (qPCR) in a 20-μl volume containing SYBR Green Universal Premix (4309155, Thermo Fisher), gene-specific primers (0.4 μM each), and 2 μl of cDNA was run on a T100 Thermal Cycler (BIO-RAD) under standardized cycling conditions: 95 °C/30 s → 40 cycles (95 °C/5 s → 60 °C/30 s). Gene expression normalized to *Gapdh* was quantified using the 2^−ΔΔCt^ method; primer specificity was validated using melt curve analysis (Table S2).

### In vitro expansion of T cells

T cells (5 × 10^6^) were resuspended in 500 μl of prewarmed PBS (37 °C) to achieve a concentration of 1 × 10^7^ cells/ml. Carboxyfluorescein succinimidyl ester (CFSE; 423801, BioLegend) was added to a final concentration of 1 μM, followed by gentle mixing and incubation in a 37 °C water bath for 10 min. To remove excess dye, 5 volumes of ice-cold RPMI 1640 complete medium were added, and the cells were placed on ice for 5 min. After centrifugation at 300*g* for 5 min, cells were washed 3 times with ice-cold RPMI 1640 complete medium. The cell pellet was resuspended in complete RPMI 1640 culture medium (supplemented with 10% FBS and 1% Pen–Strep) to 5 × 10^6^ cells/ml. Each well of a 96-well plate received 100 μl of cell suspension, followed by anti-mouse CD3/CD28 Dynabeads (11452D, Thermo Fisher) at a 4:1 cell-to-bead ratio and recombinant IL-2 (50 ng/ml, 216-16, PeproTech). The total culture volume was adjusted to 200 μl/well with additional complete medium. The plates were incubated for 72 h in a humidified incubator at 37 °C with 5% CO₂. Cell proliferation was monitored daily by phase-contrast microscopy (MODEL ECLIPSE Ts2, Nikon). On day 3, the cells were stained with PE-Cyanine7-conjugated anti-mouse CD8a (0.2 μl/sample, 100722, BioLegend) and BV421-conjugated anti-mouse CD4 (0.2 μl/sample, 100443, BioLegend) antibodies at 4 °C for 30 min. Cells were then washed with 2 ml of PBS, centrifuged at 500*g* for 5 min, and resuspended in 100 μl of PBS. The suspension was filtered through a 200-μm mesh and analyzed immediately using a flow cytometer.

### In vitro CD8^+^ T cytotoxicity assay

Mice were humanely euthanized under deep isoflurane anesthesia by cervical dislocation, and splenic and LN lymphocytes were isolated. CD8^+^ T cells were sorted using FACS (Aurora CS, Cytek), labeled with CFSE (1 μM, 10 min, 423801, BioLegend), and activated with anti-mouse CD3/CD28 Dynabeads (cell:beads ratio: 4:1), purified anti-mouse CD3ε antibody (5 μg/ml, 100340, BioLegend), and purified anti-mouse CD28 antibody (5 μg/ml, 102116, BioLegend) as described previously [48]. Activated T cells were then cocultured with B16-F10 cells (effector:target ratio: 10:1) for 48 h. Human CD8^+^ T cells were isolated from PBMCs using FACS and paired with A375 melanoma cells as target cells [19]. Tumor cell death was quantified through Annexin V/propidium iodide (PI) (KGA1105-100, KeyGEN Biotech) staining and flow cytometry.

### Western blotting

Total proteins were extracted using radioimmunoprecipitation assay (RIPA) lysis buffer (P0013B, Beyotime) supplemented with protease inhibitors (P1005, Beyotime), quantified by bicinchoninic acid‌ assay (P0009, Beyotime), and separated on 10% sodium dodecyl sulfate–polyacrylamide gel electrophoresis (SDS-PAGE) gels (20 μg per lane). Proteins were transferred to polyvinylidene difluoride membranes (120 V, 90 min, PVH00010, Merck Millipore), blocked with 5% nonfat milk (P0216, Beyotime) for 2 h, and incubated overnight at 4 °C with primary antibodies. After washing with tris-buffered saline containing 0.1% Tween 20 (TBST) (T1085, Solarbio), membranes were probed with HRP-conjugated secondary antibodies (A21020, Abbkine) and visualized using enhanced chemiluminescence (ECL) substrate (P0018S, Beyotime). Band intensities were quantified using ImageJ (v1.53e, National Institutes of Health) with glyceraldehyde-3-phosphate dehydrogenase (GAPDH) as the loading control.

### Combination therapy in the B16-F10 subcutaneous melanoma model

C57BL/6 mice were subcutaneously inoculated in the right flank with 1 × 10^5^ B16-F10 melanoma cells. HI-TOPK-032 (50 μg per mouse, HY-101550, MedChemExpress) was administered intraperitoneally every other day starting from day 3 post-inoculation. Anti-PD-1 antibody (anti-PD-1) (250 μg per mouse, GM-28206AB, Jiman Biotech) was injected intraperitoneally on days 15, 17, and 19, with the mouse immunoglobulin G1 (IgG1) isotype control antibody (250 μg per mouse, GM-77407AB, Jiman Biotech) serving as the treatment control. Tumors were excised on day 21 and measured. Single-cell suspensions from the tumors were analyzed using flow cytometry.

### Bulk RNA sequencing

WT and TOPK-KO Jurkat cells were cultured and harvested, then lysed directly in TRIzol (15596018CN, Thermo Fisher) by thorough pipetting to obtain a homogeneous lysate. The lysates were flash-frozen in liquid nitrogen and stored at −80 °C until RNA extraction. CD8^+^ T cells from WT and *Topk*^−/−^ mice were flow-sorted, activated with anti-mouse CD3/CD28 Dynabeads (30 min, 11452D, Thermo Fisher), and then lysed in TRIzol and stored at −80 °C using the same procedure as described above. RNA quality was assessed by electrophoresis on 1% agarose gel (degradation/contamination, A800342, Aladdin), NanoPhotometer (OD260/280 = 1.8 to 2.2; OD260/230 ≥ 1.8, Implen GmbH), and Qsep400 (RNA integrity number ≥ 8.0, BiOptic). Libraries were prepared with the NEBNext Ultra RNA Library Prep Kit (E7490, New England Biolabs, NEB) and sequenced on an Illumina NovaSeq X Plus platform (PE150, Illumina Inc.). Raw reads were filtered with fastp (v0.23.1) [49], aligned to GRCh38 with STAR (v2.7.10a) [50], and quantified with featureCounts (v2.0.3) [51]. Differential expression analysis (|log_2_FC| ≥ 1 and adjusted *P* < 0.05 considered important) was performed with DESeq2 (v1.36.0) [52]. Functional enrichment analysis of differentially expressed genes was conducted using clusterProfiler (v4.4.4) [53] with a false discovery rate (FDR) of <0.05. Heatmaps were generated using pheatmap (v1.0.12, https://CRAN.R-project.org/package=pheatmap).

### Single-cell RNA sequencing and data analysis

Raw human single-cell RNA-sequencing (scRNA-seq) data from 10 melanoma tumor biopsies (untreated patients) and 10 normal LNs were obtained from Gene Expression Omnibus (GEO; https://www.ncbi.nlm.nih.gov/geo/, accession nos. GSE215120 [54,55] and GSE193449) and the European Bioinformatics Institute (EMBL-EBI; https://www.ebi.ac.uk/, accession no. E-MTAB-11536) [56]). The raw reads were aligned to the GRCh38 reference genome and quantified using Cell Ranger (v7.1.0, https://www.10xgenomics.com/support/software/cell-ranger/latest).

Subsequent analysis was performed using Seurat (v4.1.1) [57]. Low-quality cells (detected genes < 200 or > 6,000; mitochondrial content > 10%) were filtered out. The filtered data were then normalized using SCTransform, followed by principal components analysis (PCA; 30 principal components) for dimensionality reduction and visualization using Uniform Manifold Approximation and Projection (UMAP). Marker gene expression was visualized on the UMAP embedding using the FeaturePlot function in Seurat (v4.1.1) [57] to support cell type annotation. Unsupervised clustering (resolution = 0.1) using the top 2,000 highly variable genes identified the major cell populations. Immune cell subsets were reclustered (resolution = 0.2) to evaluate TOPK expression. CD8^+^ T cells were stratified into TOPK^+^ and TOPK^−^ subgroups based on TOPK expression in the scRNA-seq data, with cells showing detectable expression (normalized expression > 0) defined as TOPK^+^ and those with no detectable expression (expression = 0) defined as TOPK^−^. Differential expression of the cytotoxic markers [tumor necrosis factor (*TNF*), perforin1 (*PRF1*), *GZMB*, and granzyme H (*GZMH*)] and the activation marker CD8 α chain (*CD8A*) was assessed between TOPK^+^ and TOPK^−^ CD8^+^ T cell subgroups using the Wilcoxon rank-sum test (FDR < 0.05), with the results visualized using split violin plots (ggplot2, v3.4.2, https://CRAN.R-project.org/package=ggplot2).

Tumor-bearing mice were generated by subcutaneous injection of 0.5 × 10^6^ B16-F10 melanoma cells into 6-week-old *Topk*^fl/fl^ (*n* = 3) and *Cd8*^Cre^*Topk*^fl/fl^ (*n* = 3) mice under SPF conditions. Tumors were resected on day 16 post-inoculation, mechanically dissociated, and enzymatically digested using collagenase IV (1 mg/ml; C4-BIOC, Sigma-Aldrich) at 37 °C for 30 min. The resulting single-cell suspensions were enriched for CD45^+^ immune cells using magnetic bead sorting (CD45 MicroBeads, 130-052-301, Miltenyi Biotec) and processed for 10x Genomics Chromium Single Cell 3′ v3.1 (10x Genomics) library preparation. Sequencing was performed on an Illumina NovaSeq 6000 platform (PE150, Illumina Inc.) with a median sequencing depth of 50,000 reads per cell. Raw data were processed using Cell Ranger (v7.1.0; https://www.10xgenomics.com/support/software/cell-ranger/latest). To remove batch effects between samples, we used the Harmony (v1.2.0, https://cran.r-project.org/package=harmony) pipeline and performed clustering using the Leiden clustering approach implemented in Seurat (v4.1.1, https://satijalab.org/seurat/) with standard quality control (200 to 6,000 genes per cell; mitochondrial reads < 10%). t-SNE (t-distributed stochastic neighbor embedding) dimensionality reduction was performed in Seurat (v4.1.1, https://satijalab.org/seurat/) using the first 30 principal components. After quality filtering, unsupervised clustering was performed using the top 3,000 highly variable genes. Cell clusters were annotated based on canonical marker expression as previously described [58]. Cell–cell communication networks were reconstructed using CellChat (v1.5.0, https://github.com/sqjin/CellChat) based on the built-in CellChatDB.mouse ligand–receptor interaction database. Differential expression analysis was performed in Seurat (v4.1.1, https://satijalab.org/seurat/) using the Wilcoxon rank-sum test. Genes with |log_2_FC| ≥ 1 and adjusted *P* < 0.05 were considered differentially expressed. Heatmaps were generated using pheatmap (v1.0.12; https://CRAN.R-project.org/package=pheatmap).

### Transduction of human CD8^+^ T cells

Frozen healthy donor-derived CD8^+^ T cells were obtained from Shanghai ORIBIOTECH Co. Ltd. Cryopreserved human CD8^+^ T cells were rapidly thawed in a water bath at 37 °C with gentle agitation. Upon complete thawing, cells were diluted in 5 volumes of prewarmed complete RPMI 1640 medium (supplemented with 10% FBS and 1% Pen–Strep) and centrifuged at 300*g* for 5 min. The pellet was then resuspended in fresh complete medium, and cell viability (>90%) was confirmed by trypan blue exclusion using a 0.4% trypan blue solution (C0011S, Beyotime) and a hemocytometer (C100-Pro, Ruiwode). For activation, cells were seeded in culture plates and stimulated with anti-human CD3/CD28 Dynabeads (11161D, Thermo Fisher) at a 4:1 cell-to-bead ratio in complete medium. After incubation for 24 h (37 °C, 5% CO₂), 50% of the supernatant was replaced with lentiviral particles (Vector, an empty-vector control lentivirus; TOPK-OE, a lentivirus encoding TOPK; or IRF5-OE, a lentivirus encoding IRF5; Jikai Gene Biotech) at a multiplicity of infection (MOI) of 20, supplemented with a transduction enhancer polybrene (8 μg/ml, H9268, Sigma-Aldrich) and recombinant IL-2 (50 ng/ml, 200-02, PeproTech). Following 16 h of incubation, the medium was replaced with fresh complete medium supplemented with IL-2 (50 ng/ml), and cultured for an additional 48 h. Transduced cells were sorted using FACS (Aurora CS, Cytek) based on green fluorescent protein (GFP) or mCherry expression. Sorted cell populations were then expanded for downstream functional assays.

### Phosphorylation microarray detection

Splenocytes and LN-derived cells isolated from WT and *Topk*^−/−^ mice were activated in vitro with anti-mouse CD3/CD28 Dynabeads (11452D, Thermo Fisher) for 30 min at 37 °C, followed by centrifugation (300*g*, 5 min). The cell pellets were then resuspended in 200 μl of cell lysis buffer containing protease and phosphatase inhibitors (P1045, Beyotime) ​and immediately flash-frozen in liquid nitrogen. The cell lysates were homogenized using Full Moon cell lysis magnetic beads (LB001, Full Moon BioSystems Inc.) through 5 cycles of vortexing (30 s each) and incubation on ice (10 min), followed by repeated centrifugation (12,000*g*, 4 °C, 15 min) to clarify supernatants. Protein concentration was quantified using a BCA assay, and 25 μg of protein per sample was labeled with biotin/N,N-dimethylformamide (DMF) (10 μg/μl, KAS02-Biotin, Full Moon BioSystems Inc. prepared by dissolving 1 mg of biotin reagent in 100 μl of DMF). The labeling reaction (70 μl final volume) was performed at 25 °C for 2 h and terminated with 30 μl of stop buffer; the samples were then stored at −80 °C. For hybridization, the protein microarray was equilibrated (45 min, 25 °C), blocked in 30 ml of blocking solution on an orbital shaker with gentle agitation (45 min), and incubated with biotinylated protein-coupling solution mixtures with gentle shaking (6 ml, 2 h). Post-hybridization detection involved incubation with Cy3-Streptavidin (0.5 mg/ml in detection buffer) for 20 min in the dark with shaking; all required reagents were included in the Antibody Array Assay Kit (KAS02, Full Moon BioSystems). Images were acquired using GenePix Pro 6.0 (Molecular Devices/Axon Instruments), and data normalization accounted for lateral microarray bias by separately adjusting the left/right block medians. Paired antibodies targeting 1,168 proteins were analyzed, and phosphorylation ratios for 584 sites were calculated as the ratio of phosphorylated to non-phosphorylated signals. Intersample fold changes were determined by comparing the phosphorylated/non-phosphorylated ratios between the experimental and control groups.

### Co-immunoprecipitation

Jurkat cells were lysed in 200 μl of immunoprecipitation (IP) lysis buffer (P0013, Beyotime) supplemented with protease and phosphatase inhibitors (P1045, Beyotime) for 30 min on ice. Cell lysates were clarified by centrifugation at 12,000*g*, for 15 min at 4 °C, and the supernatants were collected. Equal amounts of protein (500 μg) were incubated with 2 μg of specific antibody (anti-TOPK antibody (EPR21983, Abcam) or control IgG (P2175S-6, Beyotime) overnight at 4 °C with gentle rotation, followed by the addition of 20 μl of protein A magnetic beads (P2175S-4, Beyotime) for 2 h at 4 °C. The beads were washed 3 times with cold lysis buffer, and bound proteins were eluted by boiling in SDS loading buffer (P2175S-8, Beyotime) for 10 min. Immunoprecipitated proteins were analyzed by Western blotting using indicated antibodies.

### Statistical analysis

Experimental data were analyzed using GraphPad Prism 8 (GraphPad Software). Statistical methodologies, including specific test types and sample sizes, are detailed in the respective figure legends. All statistical analyses were conducted using 2-tailed tests, with the significance threshold (α) set at 0.05. Trend lines (line graphs) and intergroup comparisons (bar plots) display the group means; error bars denote the standard error of the mean (SEM).

## Results

### A subpopulation of infiltrating CD8^+^ T cells in melanoma exhibits TOPK expression, correlating with reduced effector function factors

**Fig. 1.**
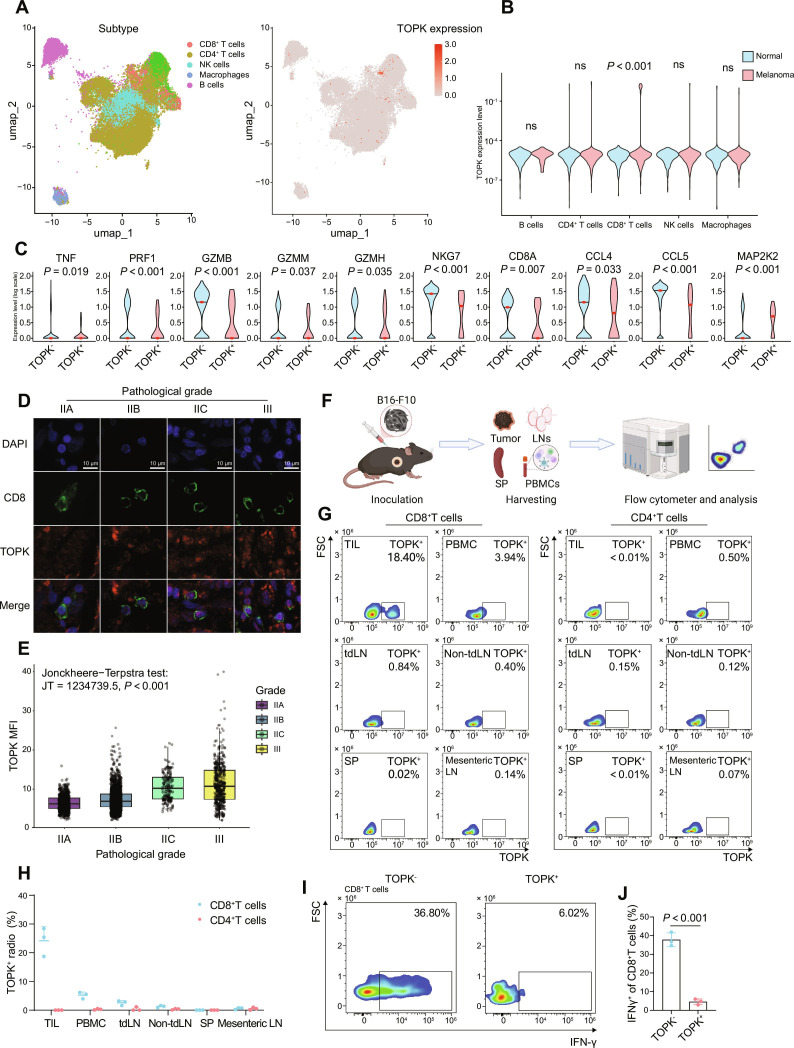
Characterization of TOPK^+^CD8^+^ T cell subsets in melanoma. (A to C) scRNA-seq analysis of lymphocytes from 10 patients with untreated melanoma (GSE215120; 16,011 cells post-quality control) and 10 normal LNs (GSE193449 and E-MTAB-11536; 23,449 cells). Datasets were integrated and jointly analyzed after merging. (A) UMAP plot colored by annotated immune subsets (left panel) and *TOPK* expression (right panel). (B) Differential expression of *TOPK* in B cells, CD4^+^ T cells, CD8^+^ T cells, and NK cells between normal LNs and melanoma tissues. (C) Violin plots showing transcript levels of indicated genes between TOPK^−^ and TOPK^+^ subsets in melanoma-infiltrating CD8^+^ T cells. mRNA expression level was quantified as log_10_(normalized counts +1). The median is indicated by the line within each violin. (D and E) Multiplex immunofluorescence analysis of TOPK expression in CD8^+^ T cells across melanoma pathological grades (*n* = 90 patients). Representative multiplex immunofluorescence images of melanoma tissue sections from 4 pathological grades (IIA, IIB, IIC, and III) (D). Nuclei, DAPI (blue); CD8^+^ T cells, anti-CD8 antibody (green); TOPK, anti-TOPK antibody (red). Scale bar, 10 μm. TOPK MFI in CD8^+^ T cells across melanoma pathological grade. (E) JT denotes the Jonckheere–Terpstra test statistic (rank-based) that quantifies the strength of a monotonic trend across ordered groups. Data represent 2,398 CD8^+^ T cells from 90 patients: IIA (*n* = 627), IIB (*n* = 1,262), IIC (*n* = 138), III (*n* = 371). (F to J) C57BL/6 mice (*n* = 3) were inoculated with 0.5 × 10^6^ B16-F10 cells. Tissues (tumor, tdLN, non-tdLN, mesenteric LN, and SP) and PBMCs were harvested on day 16 since tumor inoculation for flow cytometry analysis. Workflow of experiment process (F) and representative flow cytometry plots (G) and quantification (H) of TOPK expression in CD8^+^ and CD4^+^ T cells. Representative flow cytometry plots (I) and quantification (J) of the proportion of IFN-γ^+^ of CD8^+^ T cells. Data are shown as violin plots (B and C), medians with interquartile range (IQR) (E), and mean ± SEM (H and J). Statistical significance was assessed using an unpaired 2-tailed *t* test (B, C, and J) and Jonckheere–Terpstra test (E). DAPI, 4′,6-diamidino-2-phenylindole; TOPK, T-LAK cell-originated protein kinase; TIL, tumor-infiltrating lymphocyte; LN, lymph node; NK cell, natural killer cell; PBMC, peripheral blood mononuclear cell; tdLN, tumor draining lymph node; Non-tdLN, non-tumor-draining lymph node; SP, spleen; mesenteric; LN, mesenteric lymph node; MFI, mean fluorescence intensity; IFN-γ, interferon-γ; umap, Uniform manifold approximation and projection.

Previous studies have demonstrated that TOPK is highly expressed in various cancers and acts as a MAPK, promoting the malignant proliferation of cancer cells [37,38,42]. Here, we analyzed scRNA-seq data obtained from the GEO database (GSE215120 [54,55] and GSE193449) and the ArrayExpress database at EMBL-EBI (E-MTAB-11536) [56], including 10 melanoma tumor biopsies from untreated patients and 10 normal LN samples from healthy individuals, with the latter serving as controls (Fig. S3). We identified a subset of TOPK^+^CD8^+^ T cells within the TILs of patients with melanoma, compared with CD8^+^ T cells from normal LNs of healthy individuals. Low-level TOPK expression was detectable in CD4^+^ T cells, natural killer (NK) cells, and macrophages; however, no significant differences were observed between normal LNs and melanoma in these populations. Only CD8^+^ T cells showed a significant increase in TOPK expression in melanoma compared with normal LNs (Fig. 1A and B). Further analysis of the transcriptional differences between TOPK^+^CD8^+^ T cells and TOPK^−^CD8^+^ T cells revealed that the former exhibited significantly lower mRNA abundance of effector functions [*TNF, PRF1, GZMB,* granzyme M (*GZMM*)*, GZMH,* and natural killer cell granule protein 7 (*NKG7*)], co-receptor (*CD8A*), and chemokine-related factors [C–C motif chemokine ligand 4 (*CCL4*) and C–C motif chemokine ligand 5 (CCL5)] compared with those in the TOPK^−^ subset (Fig. 1C). However, inhibitory factors associated with suppressed effector functions (MAP2K2) [59] were markedly elevated in the TOPK^+^CD8^+^ T cell subgroup compared with the TOPK^−^ subgroup (Fig. 1C). These results suggest a potential link between high TOPK expression in melanoma-infiltrating CD8^+^ T cells and diminished activation, chemotaxis, and effector functions.

To determine whether TOPK expression in melanoma-infiltrating CD8^+^ T cells varies across pathological grades, we performed multiplexed immunofluorescence staining for CD8 and TOPK proteins in patient-derived melanoma tissue microarrays.​ TOPK expression in infiltrating CD8^+^ T cells showed a significant increasing trend with higher pathological grades (Fig. 1D and E). This suggested that TOPK expression in tumor-infiltrating CD8^+^ T cells may serve as a potential indicator of melanoma pathological grading. Subsequently, we established a subcutaneous tumor model in mice using the malignant melanoma cell line B16-F10. We found that TOPK was enriched in tumor-infiltrating CD8^+^ T cells, whereas TOPK expression of CD8^+^ T cells from PBMCs, tumor-draining LNs, non-tumor-draining LNs, mesenteric LNs, and SP was low and detected only in a small fraction of cells (Fig. 1F to H). Additionally, a few of TOPK^+^CD4^+^ T cell subsets were observed across the same sample types (PBMCs, tumor-draining LNs, non-tumor-draining LNs, mesenteric LNs, and SP) (Fig. 1F to H). ​Importantly, we detected a significantly lower capacity to produce IFN-γ in TOPK^+^CD8^+^ T cells compared with TOPK^−^CD8^+^ T cells (Fig. 1I and J). Our findings thus suggest that TOPK expression in tumor-infiltrating CD8^+^ T cells may be associated with diminished immune functionality in the melanoma TME.

### Absence of TOPK enhances the antitumor effector functions of CD8^+^ T cells

To investigate the role of TOPK in CD8^+^ T cells, *Topk* global knockout mice (*Topk*^−/−^) and CD8^+^ T cell conditional knockout mice (*Cd8*^Cre^*Topk*^fl/fl^) were obtained and used in this study. Genomic deletion of *Topk* was validated using PCR and agarose gel electrophoresis (Fig. S4A to F). Compared with those of WT mice, the sizes of the SPs, LNs, and other major organs of *Topk*^−/−^ mice showed no significant differences (Fig. S4G). However, compared with WT, the LNs of *Topk*^−/−^ mice showed a reduced proportion of CD19^+^ cells among CD45^+^ cells, together with an increased proportion of CD3^+^CD4^+^ cells and a decreased proportion of CD3^+^CD8^+^ cells among CD45^+^ cells. In PBMCs, *Topk*^−/−^ mice exhibited a decreased proportion of CD19^+^ cells and an increased proportion of CD3^+^CD4^+^ cells among CD45^+^ cells. In the SP, the proportion of TCRγδ^+^CD3^+^ cells among CD45^+^ cells were increased in *Topk*^−/−^ mice (Fig. S4H). Interestingly, in the subcutaneous tumor mouse model established using the malignant melanoma cell line B16-F10, the tumor growth rates, volumes, and weights in *Topk*^−/−^ mice were significantly lower than those in WT mice (Fig. S5A to D).

**Fig. 2.**
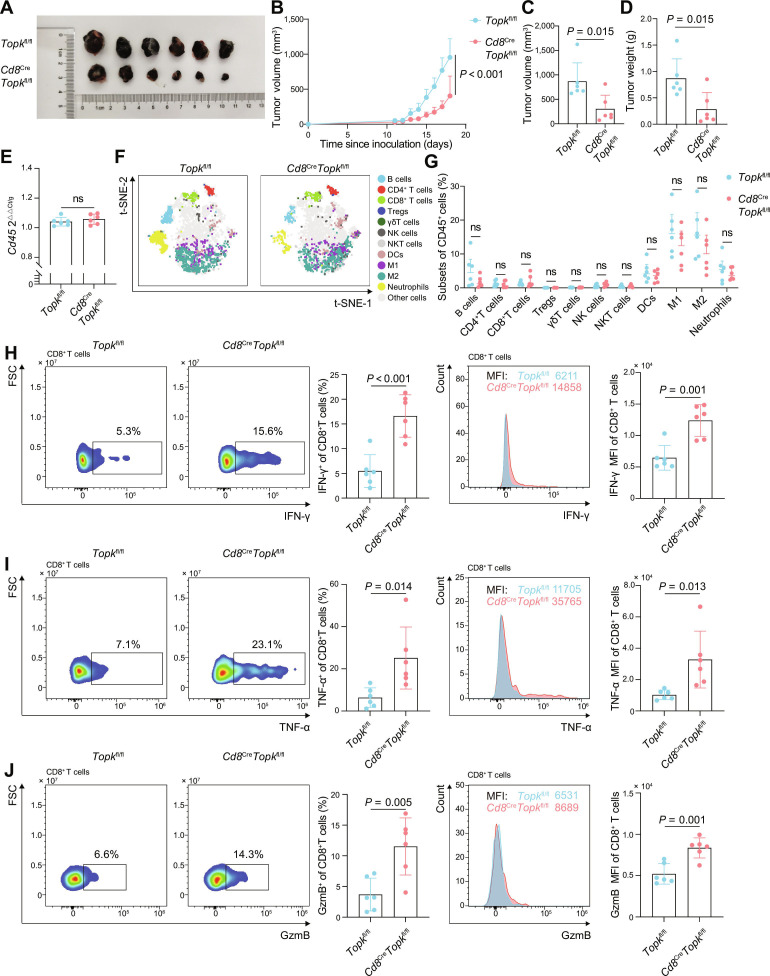
Impact of *Topk* deletion in CD8^+^ T cells on antitumor immunity. *Cd8*^Cre^*Topk*^fl/fl^ mice and *Topk*^fl/fl^ littermate controls (*n* = 6 per group) were inoculated subcutaneously with 0.5 × 10^6^ B16-F10 cells. (A to D) Photographs of the tumor collected at day 18 since inoculation (A), tumor growth kinetics (B), tumor volumes at day 18 since inoculation (C), and tumor weights at day 18 since inoculation (D) in *Cd8*^Cre^*Topk*^fl/fl^ versus *Topk*^fl/fl^ mice. (E) Cd45 mRNA expression levels in tumor tissues acquired from *Topk*^fl/fl^ and *Cd8*^Cre^*Topk*^fl/fl^ mice were quantified using qPCR (normalized to *Gapdh*). (F and G) t-SNE visualization (F) and quantification data (G) of TIL subsets in tumor tissues from *Topk*^fl/fl^ and *Cd8*^Cre^*Topk*^fl/fl^ mice, based on flow cytometry data. (H to J) Frequency (left) and MFI (right) of IFN-γ (H), TNF-α (I), and GzmB (J) in CD8^+^ T cells across each group, shown as representative flow plots/histograms and quantification. Data represent the mean ± SEM (B to E and G to J). Mixed-effects model with group and time as fixed effects and individual mice as random effects (B), unpaired 2-tailed *t* test (C to E and G to J). ns: not significant. *Topk*^fl/fl^, *Topk* floxed mouse; *Cd8*^Cre^*Topk*^fl/fl^, CD8-specific *Topk* conditional knockout mouse; qPCR, quantitative real-time polymerase chain reaction; TIL, tumor-infiltrating lymphocyte; t-SNE, t-distributed stochastic neighbor embedding; IFN-γ, interferon-γ; TNF-α, tumor necrosis factor α; GzmB, granzyme B; SEM, standard error of the mean; *Gapdh*, glyceraldehyde-3-phosphate dehydrogenase; Treg, regulatory T cell; NK cell, natural killer cell; NKT cell, natural killer T cell; DC, dendritic cell; M1/M2, macrophage type 1/2; Neutrophil, neutrophil granulocyte.

To confirm that TOPK deficiency suppresses tumor growth mediated by CD8^+^ T cell-dependent mechanisms, we established subcutaneous tumor models in *Cd8^Cre^Topk*^fl/fl^ and *Topk*^fl/fl^ mice. Consistent with our findings in the *Topk*^−/−^ mice compared with WT mice, conditional *Cd8*^Cre^*Topk*^fl/fl^ mice exhibited significantly reduced tumor growth rates, volumes, and weights compared with *Topk*^fl/fl^ controls (Fig. 2A to D). To determine whether the absence of TOPK influences immune cell infiltration in tumors, we measured the *Cd45* mRNA abundance in tumors using qPCR. Quantitative analysis revealed no significant differences in *Cd45* mRNA abundance between *Topk*^fl/fl^ and *Cd8*^Cre^*Topk*^fl/fl^ groups, or between the WT and *Topk*^−/−^ mice (Fig. 2E and Fig. S5E). Flow cytometry further demonstrated comparable compositions and frequencies of tumor-infiltrating immune cells in the *Topk*^fl/fl^ and *Cd8*^Cre^*Topk*^fl/fl^ mice, as well as in WT and *Topk*^−/−^ mice (Fig. 2F and G and Fig. S5F and G).

Building on the above findings that​ TOPK was specifically enriched in tumor-infiltrating CD8^+^ T cells and that both *Topk*^−/−^ and *Cd8*^Cre^*Topk*^fl/fl^ mice exhibited comparable tumor suppression phenotypes, we investigated the impact of TOPK deficiency on tumor-infiltrating CD8^+^ T cells. Compared with WT controls, tumor-infiltrating CD8^+^ T cells from *Topk*^−/−^ mice exhibited significantly enhanced secretion of effector cytokines (IFN-γ and TNF-α) and cytolytic molecules GzmB, accompanied by elevated CD69 expression, indicative of enhanced activation (Fig. S5H to K). In contrast, the production of granzyme A (GzmA) and perforin did not differ significantly between *Topk*^−/−^ and WT CD8^+^ TILs (Fig. S6A and B). Similarly, *Cd8*^Cre^*Topk*^fl/fl^ mice showed comparable increases in IFN-γ^+^, TNF-α^+^, and GzmB^+^CD8^+^ TILs relative to those in the *Topk*^fl/fl^ littermates (Fig. 2H to J), confirming that TOPK loss in CD8^+^ T cells was enough to enhance their effector function. The expression levels of costimulatory receptors, including cluster of differentiation 28 (CD28) and cluster of differentiation 226 (CD226), and immune checkpoint molecules [cluster of differentiation 96 (CD96), PD-1, CTLA-4, LAG-3, T cell immunoglobulin and mucin domain-containing protein 3 (TIM-3), and TIGIT] were comparable between the 2 groups (Fig. S6C). However, when compared with WT controls, *Topk*^−/−^ mice showed decreased frequencies of CD19^+^ cells and increased frequencies of CD3^+^CD4^+^ cells among CD45^+^ cells in the LNs, accompanied by a reduced frequency of CD11b^+^CD11c^+^ cells. In PBMCs, *Topk*^−/−^ mice exhibited decreased CD19^+^ cells and increased CD3^+^CD4^+^ cells among CD45^+^ cells, as well as CD3^+^CD8^+^ cells among CD45^+^ cells. In the SP, *Topk*^−/−^ mice displayed increased CD19^+^ cells and decreased CD3^+^CD8^+^ cells and CD11b^+^CD11c^+^ cells among CD45^+^ cells (Fig. S6D). In contrast, *Cd8*^Cre^*Topk*^fl/fl^ mice exhibited no significant differences in GzmA or perforin production, costimulatory receptor, or immune checkpoint molecule expression in CD8^+^ TILs compared with those in the *Topk*^fl/fl^ littermates (Fig. S7A to C). Moreover, no changes in immune cell frequencies were observed in the LNs, PBMCs, or SP of *Cd8*^Cre^*Topk*^fl/fl^ mice, suggesting that TOPK may exert cell type-specific roles beyond CD8^+^ T cells (Fig. S7D). Furthermore, consistent with the findings in melanoma, inoculation of the murine colorectal cancer cell line MC38 into *Topk*^fl/fl^ and *Cd8*^Cre^*Topk*^fl/fl^ mice demonstrated that TOPK deficiency similarly enhanced antitumor responses (Fig. S8). In summary, our findings suggest that the absence of TOPK in CD8^+^ T cells has significantly enhanced their production of effector cytokines, which contributes to reduced tumor growth in a melanoma model, without significantly affecting overall immune cell infiltration.

### TOPK deficiency alters the transcriptional profile of tumor-infiltrating CD8^+^ T cells

**Fig. 3.**
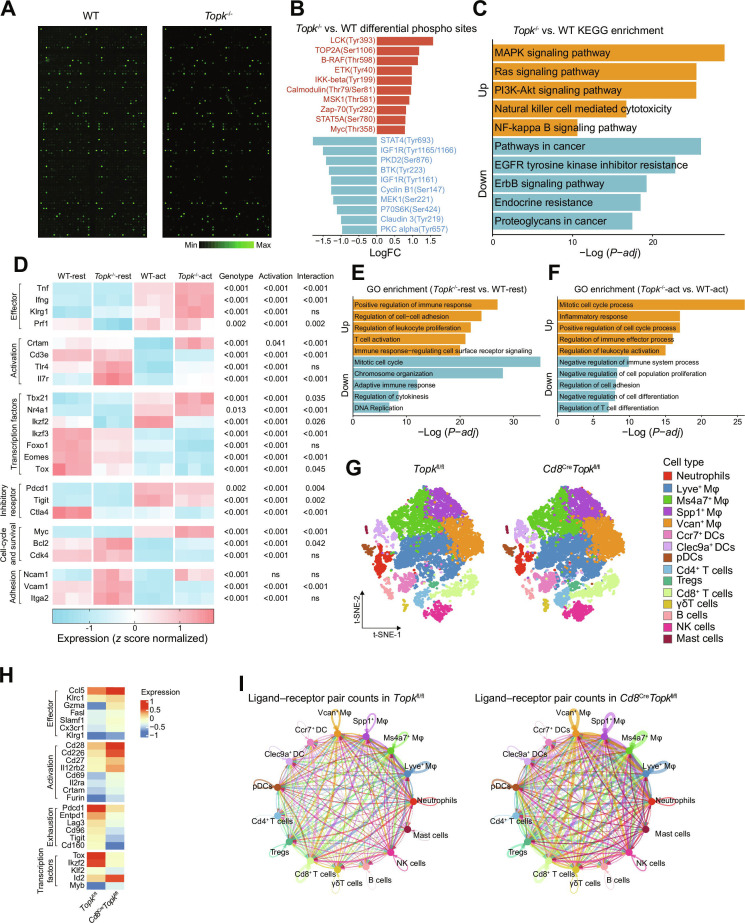
*Topk* deficiency remodels phosphorylation and transcriptional profiles of CD8^+^ T cells. (A to C) Targeted phospho-antibody microarray analysis of ex vivo-activated CD8^+^ T cells from WT or *Topk*^−/−^ mice after anti-CD3/CD28 Dynabead simulation for 30 min (*n* = 3 per group). (A) Representative microarray images; green fluorescence intensity corresponds to phosphorylation levels. (B) Differential phosphorylation sites in *Topk*^−/−^ versus WT CD8^+^ T cells (top 10 up-regulated and top 10 down-regulated sites (|logFC| > 0.5). (C) KEGG pathway enrichment of altered phospho-proteins. (D to F) RNA-seq analysis was performed on unstimulated (resting) rand anti-CD3/CD28 bead-activated (30 min) CD8^+^ T cells isolated from WT and *Topk*^−/−^ mice (*n* = 3 per group). Differential expression of genes associated with 6 functional modules (Effector, Activation, Transcription factors, Inhibitory receptors, Cell cycle and survival, and Adhesion) in tumor-infiltrating CD8^+^ T cells. Heatmap of differentially expressed genes (*z* scores normalized); the last 3 columns show *P* values from a mixed-effects model with genotype (*Topk*^−/−^ versus WT) and activation status (activated versus rest) as fixed effects, a genotype × activation interaction term, and mouse as a random intercept. (D). GO Biological Process enrichment analysis of genes differentially expressed between *Topk*^−/−^ and WT CD8^+^ T cells under resting (E) and activation (F). (G to I) Single-cell compositional analysis of tumor-infiltrating immune cells in tumor tissues derived from *Topk*^fl/fl^ and *Cd8^Cre^Topk^fl/fl^* mice (*n* = 3 per group). (G) t-SNE projection of TIL subsets. (H) Differential expression of genes associated with 4 functional modules (Effector, Activation, Exhaustion, and Transcription factors) in tumor-infiltrating CD8^+^ T cells. (I) Cell–cell communication networks reconstructed using CellChat (v1.6.1). Edge width indicates the number of ligand–receptor pairs between cell types. (D) Mixed-effects model with genotype and activation as fixed effects and sample as a random intercept. WT, wild type; *Topk*^−/−^, *Topk* global knockout; CD3/CD28, cluster of differentiation 3 and 28; FC, fold change; KEGG, Kyoto Encyclopedia of Genes and Genomes; RNA-seq, RNA sequencing; GO, Gene Ontology; t-SNE, t-distributed stochastic neighbor embedding; TIL, tumor-infiltrating lymphocyte; CellChat, cell–cell communication analysis software; MFI, mean fluorescence intensity; SEM, standard error of the mean; act, activated; Neutrophil, neutrophil granulocyte; Mφ, macrophage; DC, dendritic cell; Treg, regulatory T cell; NK cell, natural killer cell; *P*-*adj*, adjusted *P* value.

First, we examined the TOPK expression levels in murine CD8^+^ T cells at multiple time points following in vitro activation. We observed that TOPK positivity peaked at 30-min post-activation (Fig. S9). Therefore, cells were harvested at 30-min post-activation for subsequent analyses. ​Previous studies identified TOPK as a serine/threonine kinase [60,61]. To delineate its role in CD8^+^ T cell effector programs, we performed phosphorylation microarray analysis on in vitro-activated CD8^+^ T cells isolated from WT and *Topk*^−/−^ mice. TOPK deficiency was associated with increased phosphorylation of key proteins governing T cell activation and effector function, including LCK, ZAP-70, IKK-β, and Myc (Fig. 3A and B). Kyoto Encyclopedia of Genes and Genomes (KEGG) pathway enrichment analysis of differentially phosphorylated molecules revealed the up-regulation of the MAPK, Ras, and phosphatidylinositol 3-kinase (PI3K)-AKT signaling pathways, whereas pathways in cancer and epidermal growth factor receptor (EGFR) tyrosine kinase inhibitor resistance signaling were down-regulated (Fig. 3C). These results suggest that TOPK ablation potentiates CD8^+^ T cell activation by amplifying activation-related signaling and effector pathways.

We next performed RNA-seq and mixed-effects modeling to assess the transcriptional impact of *Topk* deficiency on CD8^+^ T cells under resting and activated conditions. We used a mixed-effects model to quantify the independent and interactive effects of genotype (WT versus *Topk*^−/−^) and activation status (rest versus activated) on gene expression, including a genotype × activation interaction term. Biological replicate (mouse) was modeled as a random effect to account for interindividual variability. The genotype *P* value tests the overall difference between WT and *Topk*^−/−^ across both conditions; the activation *P* value tests the overall difference between resting and activated states across genotypes; and the interaction *P* value (genotype × activation) tests whether the effect of activation differs by genotype and whether *Topk* deficiency alters the activation-induced transcriptional change. Effector genes, including *Tnf,* interferon-γ (*Ifng*), killer cell lectin-like receptor subfamily G member 1 (*Klrg1*), and *Prf1*, showed significant main effects of genotype and activation (Fig. 3D). Interaction effects were significant for *Tnf*, *Ifng*, and *Prf1* but not *Klrg1*, indicating that *Topk* deficiency selectively amplifies activation-driven induction of a subset of effector transcripts. Activation-associated genes, including cytotoxic and regulatory T cell molecule (*Crtam*), CD3e molecule, ε chain of T cell receptor complex (*Cd3e*), toll-like receptor 4 (*Tlr4*), and IL-7 receptor (*Il7r*), likewise showed significant effects of genotype and activation (Fig. 3D). Interaction effects were significant for *Crtam*, *Cd3e*, and *Il7r* but not *Tlr4*, indicating that *Topk* deficiency selectively enhances activation-driven induction of specific activation-related transcripts. Pro-effector transcription factors, including T-box transcription factor 21 (*Tbx21*) and nuclear receptor subfamily 4 group A member 1 (*Nr4a1*), showed significant genotype, activation, and genotype × activation effects, with higher expression in *Topk*^−/−^ CD8^+^ T cells (Fig. 3D). Conversely, inhibitory/dysfunction-associated transcription factors, including IKAROS family zinc finger 2 (*Ikzf2*), IKAROS family zinc finger 3 (*Ikzf3*), forkhead box O1 (*Foxo1*), eomesodermin (*Eomes*), and thymocyte selection-associated high mobility group box (*Tox*), showed significant genotype and activation effects and were reduced in *Topk*^−/−^ cells; interaction effects were significant for all except *Foxo1*. Inhibitory receptor genes, including programmed cell death 1 (*Pdcd1*), *Tigit,* and cytotoxic T-lymphocyte-associated protein 4 (*Ctla4*), showed significant genotype, activation, and genotype × activation effects (Fig. 3D), with reduced expression in *Topk*^−/−^ CD8^+^ T cells. Cell cycle and survival regulators—MYC proto-oncogene (*Myc*) (proliferation), BCL2 apoptosis regulator (*Bcl2*; cell survival), and cyclin-dependent kinase 4 (*Cdk4*; cell cycle progression)—showed significant genotype and activation effects (Fig. 3D), with higher expression in *Topk*^−/−^ CD8^+^ T cells; interaction effects were significant for *Myc* and *Bcl2* but not *Cdk4*. Adhesion-related genes, including neural cell adhesion molecule 1 (*Ncam1*)*,* vascular cell adhesion molecule 1 (*Vcam1*), and integrin subunit α 2 (*Itga2*), showed significant genotype effects, with higher expression in *Topk*^−/−^ CD8^+^ T cells (Fig. 3D). Activation effects were significant for *Vcam1* and *Itga2* but not *Ncam1*. Interaction effects were significant for *Vcam1* only, whereas *Ncam1* and *Itga2* showed no significant genotype × activation interaction (Fig. 3D). Collectively, these data indicate that TOPK acts as a brake on CD8^+^ T cell activation, and its loss shifts activated CD8^+^ T cells toward a more cytotoxic, less dysfunctional transcriptional state. We then performed Gene Ontology (GO) enrichment analysis of differentially expressed genes and found that, compared to the WT-rest group, the *Topk*^−/−^-rest group exhibited significant up-regulation in pathways such as “positive regulation of immune response” and “regulation of cell–cell adhesion” (Fig. 3E). Furthermore, under activated conditions, the *Topk*^−/−^ group showed increased activation of cell cycle-related pathways (Fig. 3F). Collectively, these findings suggest that TOPK-deficient CD8^+^ T cells exhibit enhanced effector functions, which may contribute to more robust antitumor responses.

To further investigate the functional consequences of TOPK deletion in tumor-specific contexts, we performed scRNA-seq on TILs isolated from *Topk*^fl/fl^ controls and *Cd8*^Cre^*Topk*^fl/fl^ mice (Fig. S10A). The cells were clustered into 15 transcriptionally distinct immune subsets using canonical lineage markers (Fig. S10B). *Cd8*^Cre^*Topk*^fl/fl^ tumors showed no significant difference in proportions among distinct immune cell subsets (Fig. 3G and Fig. S10C) but exhibited significant up-regulation of effector molecules [*Ccl5*, killer cell lectin-like receptor subfamily C member 1 (*Klrc1*), Fas ligand (*Fasl*), and signaling lymphocytic activation molecule family member 1 (*Slamf1*)], activation markers [*Cd28*, *Cd226*, cluster of differentiation 27 (*Cd27*), and IL-2 receptor subunit α (*Il2ra*)], and transcription factors promoting effector differentiation [inhibitor of DNA binding 2 (*Id2*) and MYB proto-oncogene (*Myb*)] in tumor-infiltrating CD8^+^ T cells compared to that in the controls (Fig. 3H). Conversely, exhaustion markers [*Pdcd1*, ectonucleoside triphosphate diphosphohydrolase 1 (*Entpd1*), *Lag3*, cluster of differentiation *96* (*Cd96*), *Tigit*, and *Cd160*] and transcription factors associated with dysfunction [*Tox*, *Ikzf2*, and Kruppel-like factor 2 (*Klf2*)] were markedly reduced (Fig. 3H). Cell–cell interaction analysis demonstrated pan-immune crosstalk amplification in *Cd8*^Cre^*Topk*^fl/fl^ tumors, with globally enhanced interaction probabilities across T cell, dendritic cell, macrophage, and neutrophil subsets compared to those in the *Topk*^fl/fl^ controls (Fig. 3I and Fig. S10D), indicative of a more immunologically active TME.

Together, TOPK deficiency amplifies activation-related signaling (LCK and ZAP-70) and downstream pathways (PI3K-AKT and MAPK), driving transcriptional reprogramming of CD8^+^ T cells toward enhanced effector differentiation while suppressing exhaustion-associated programs. These mechanistic insights position TOPK as a critical checkpoint limiting CD8^+^ T cell functionality in tumor immunity.

### TOPK deficiency enhances the in vitro cytotoxic function and adoptive therapy performance of CD8^+^ T cells

**Fig. 4.**
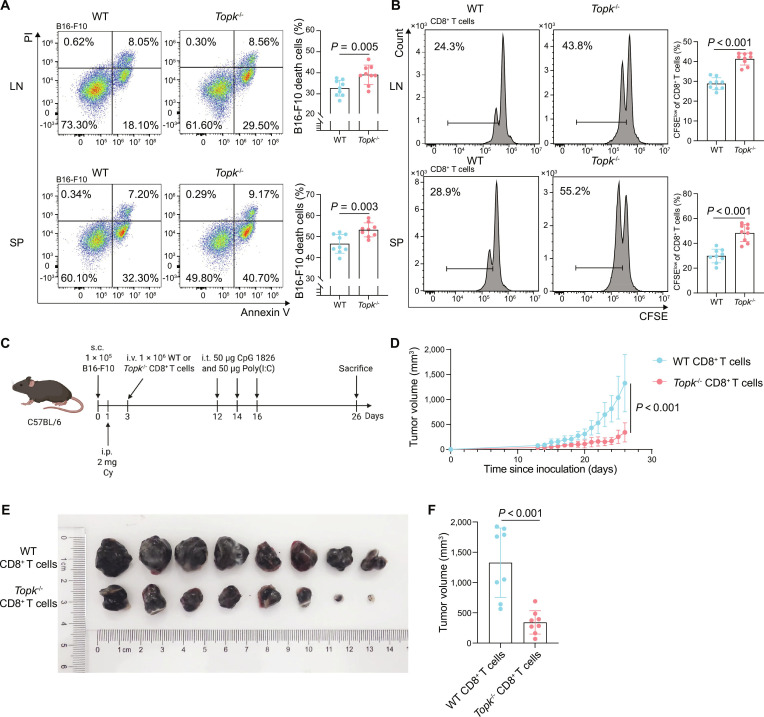
Functional and therapeutic traits of *Topk*-deficient CD8^+^ T cells in tumor models. (A and B) B16-F10 melanoma cells were cocultured with CD8^+^ T cells isolated from LN and SP of WT and *Topk*^−/−^ mice for 48 h at an effector-to-target ratio of 10:1 (*n* = 9 per group). (A) Representative flow cytometry plots (left) and quantitative analysis (right) of CD8^+^ T cell-mediated B16-F10 cell death in each group, assessed by Annexin V/PI staining. (B) Representative CFSE dilution histograms and quantification of proliferating CD8^+^ T cells from the LN or SP in each group. (C to F) C57BL/6 mice bearing subcutaneous B16-F10 tumors were subjected to adoptive transfer of WT or *Topk*^−/−^ CD8^+^ T cells (*n* = 8 per group). ​Subcutaneous melanoma models were established by subcutaneous injection of B16-F10 cells, followed by cyclophosphamide preconditioning, adoptive transfer of CD8^+^ T cells from WT or *Topk*^−/−^ mice, intratumoral CpG 1826 and poly(I:C), and daily tumor volume monitoring until sacrifice. (C) Tumor growth kinetics (D), photographs of tumors collected on day 26 post-inoculation (E), and tumor volumes on day 26 post-inoculation (F) in mice treated with WT or *Topk*^−/−^ CD8^+^ T cells. Data represent the mean ± SEM (A, B, D, and F). Unpaired 2-tailed *t* test (A, B, and F). Mixed-effects model with group and time as fixed effects and individual mice as random effects (D). WT, wild type; *Topk*^−/−^, *Topk* global knockout; LN, lymph node; SP, spleen; SEM, standard error of the mean; PI, propidium iodide; CFSE, carboxyfluorescein succinimidyl ester; Cy, cyclophosphamide; CpG 1826, CpG oligodeoxynucleotide 1826; Poly(I:C), polyinosinic:polycytidylic acid; s.c., subcutaneous injection; i.p., intraperitoneal injection; i.v., intravenous injection; i.t., intratumoral injection.

To further validate these in vivo findings and elucidate the specific effects of TOPK deficiency on CD8^+^ T cell functionality, we evaluated the cytotoxic activity and proliferative capacity of CD8^+^ T cells derived from *Topk*^−/−^ and WT mice in vitro. The results demonstrated that CD8^+^ T cells derived from either the LNs or SPs in the *Topk*^−/−^ group exhibited significantly higher cytotoxic activity against B16-F10 cells and enhanced proliferative capacity in vitro, compared with those in the WT mice (Fig. 4A and B). To further investigate the therapeutic potential of these cells, we conducted adoptive transfer experiments following a protocol described by Braun et al. [47]. The findings revealed that mice receiving *Topk*^−/−^-derived CD8^+^ T cells exhibited significantly slower tumor growth and smaller tumor volumes than did those receiving WT-derived CD8^+^ T cells (Fig. 4C to F). These results demonstrate that TOPK deficiency enhances CD8^+^ T cell cytotoxic activity in vitro and in vivo, and their proliferation in vitro.

### TOPK modulates CD8^+^ T cell function via IRF5

**Fig. 5.**
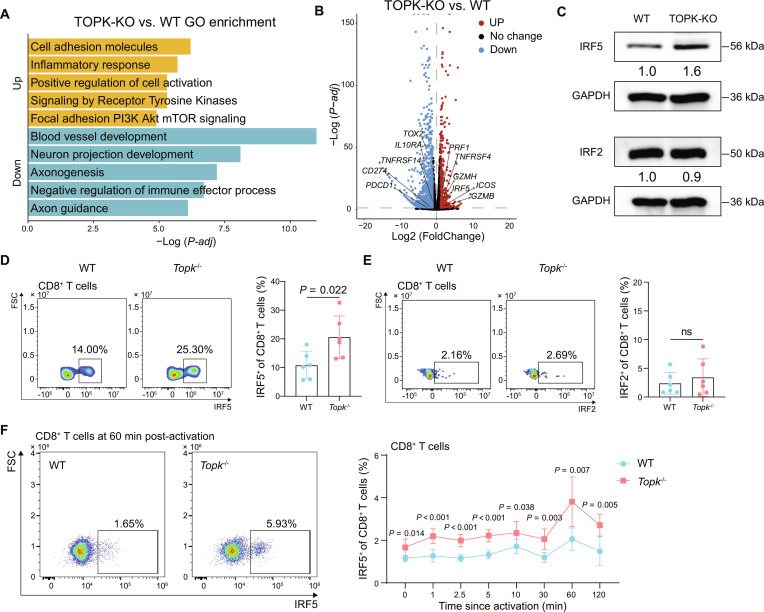
IRF5 expression in TOPK-deficient CD8^+^ T cells. (A and B) RNA-seq analysis was performed on WT and TOPK-KO Jurkat cells. Data represent 3 independent experimental runs. GO Biological Process enrichment analysis (A) and volcano plot (B) of differentially expressed genes (|log_2_FC| ≥ 1 and adjusted *P* < 0.05 considered significant) in TOPK-KO versus WT Jurkat cells. (C) Western blotting was performed to assess IRF5 and IRF2 expression levels in WT and TOPK-KO Jurkat cells. (D and E) *Topk*^−/−^ and WT mice were inoculated subcutaneously with 0.5 × 10^6^ B16-F10 cells, respectively (*n* = 6 per group). Representative flow cytometry plots (left) and quantitative analysis (right) of the frequency of IRF5^+^ (D) and IRF2^+^ (E) subsets in CD8^+^ T cells from *Topk*^−/−^ and WT mice. (F) WT and *Topk*^−/−^-derived CD8^+^ T cells were stimulated with anti-CD3/CD28 Dynabeads, and IRF5 expression was assessed by flow cytometry (*n* = 6 per group). Representative flow cytometry plots (60 min post-activation, left) and quantitative analysis (right) of the frequency of IRF5^+^ subsets in CD8^+^ T cells from *Topk*^−/−^ and WT mice at the indicated time points since activation. Data represent the mean ± SEM (D to F). Unpaired 2-tailed *t* test (D to F). ns: not significant. WT, wild type; KO, knockout; IRF, interferon regulatory factor; SEM, standard error of the mean; GAPDH, glyceraldehyde-3-phosphate dehydrogenase; min, minute; TOX2, TOX high mobility group box family member 2; IL10RA, interleukin-10 receptor subunit α; TNFRSF14, tumor necrosis factor receptor superfamily member 14; CD274, cluster of differentiation 274; PDCD1, programmed cell death 1; PRF1, perforin 1; TNFRSF4, tumor necrosis factor receptor superfamily member 4; IRF5, interferon regulatory factor 5; GZMH, granzyme H; ICOS, inducible T cell costimulatory; GZMB, granzyme B.

To investigate the molecular mechanisms by which TOPK regulates CD8^+^ T cell function, we performed RNA-seq analysis on TOPK-KO and WT Jurkat cells (Fig. S11). The results demonstrated that in TOPK-KO cells, pathways associated with cell adhesion molecules, inflammatory response, and positive regulation of cell activation were significantly up-regulated (Fig. 5A). Notably, IRF5, a transcription factor critical for CD8^+^ T cell effector function [45,62], was expressed at higher levels in TOPK-KO Jurkat cells than in WT controls (Fig. 5B).

Western blotting conducted to validate these findings at the protein level revealed increased IRF5 protein expression in TOPK-KO Jurkat cells, whereas IRF2, another family member with important roles in CD8^+^ T cell function, showed a slight decrease [63] (Fig. 5C). Flow cytometry further confirmed our results: Tumor-infiltrating CD8^+^ T cells from *Topk*^−/−^ mice exhibited a higher proportion of IRF5^+^ cells compared with those in the WT counterparts (Fig. 5D). Notably, this effect was specific to IRF5 in CD8^+^ T cells, as no significant differences were detected in IRF2 expression within CD8^+^ T cells, nor in the expression level of IRF5 or IRF2 in tumor-infiltrating CD4^+^ T cells and Tregs (Fig. 5E and Fig. S12A to D). Additionally, during in vitro activation, CD8^+^ T cells derived from *Topk*^−/−^ mice consistently exhibited higher IRF5 expression than those from WT mice (Fig. 5F and Fig. S12E). ​Together, these results show an increase in IRF5 expression in TOPK-KO versus WT CD8^+^ T cells, supporting an association between TOPK deficiency and IRF5 up-regulation. To further elucidate the mechanism by which TOPK regulates IRF5 expression, qPCR analysis was conducted and revealed that TOPK deletion increased IRF5 mRNA levels (Fig. S13A). Co-immunoprecipitation (Co-IP) assays showed no direct interaction between TOPK and IRF5 (Fig. S13B), suggesting an indirect regulatory mechanism. As TOPK lacks intrinsic DNA-binding capacity, we next assessed several known transcriptional regulators of IRF5, including tumor protein p53 (p53), RELA proto-oncogene, nuclear factor κB subunit p65 (NF-κB p65), specificity protein 1 (SP1), Jun proto-oncogene (JUN), and Spi-1 proto-oncogene (SPI1) [64–67], and identified that p53 and NF-κB p65 expression was increased in TOPK-deficient cells (Fig. S13C). However, Co-IP assays detected no direct binding between TOPK and p53 or NF-κB p65 (Fig. S13D). Collectively, these data suggest that TOPK may indirectly modulate IRF5 expression by influencing the expression of its upstream transcriptional regulators.

### TOPK suppresses effector functions by inhibiting IRF5 expression in human CD8^+^ T cells

**Fig. 6.**
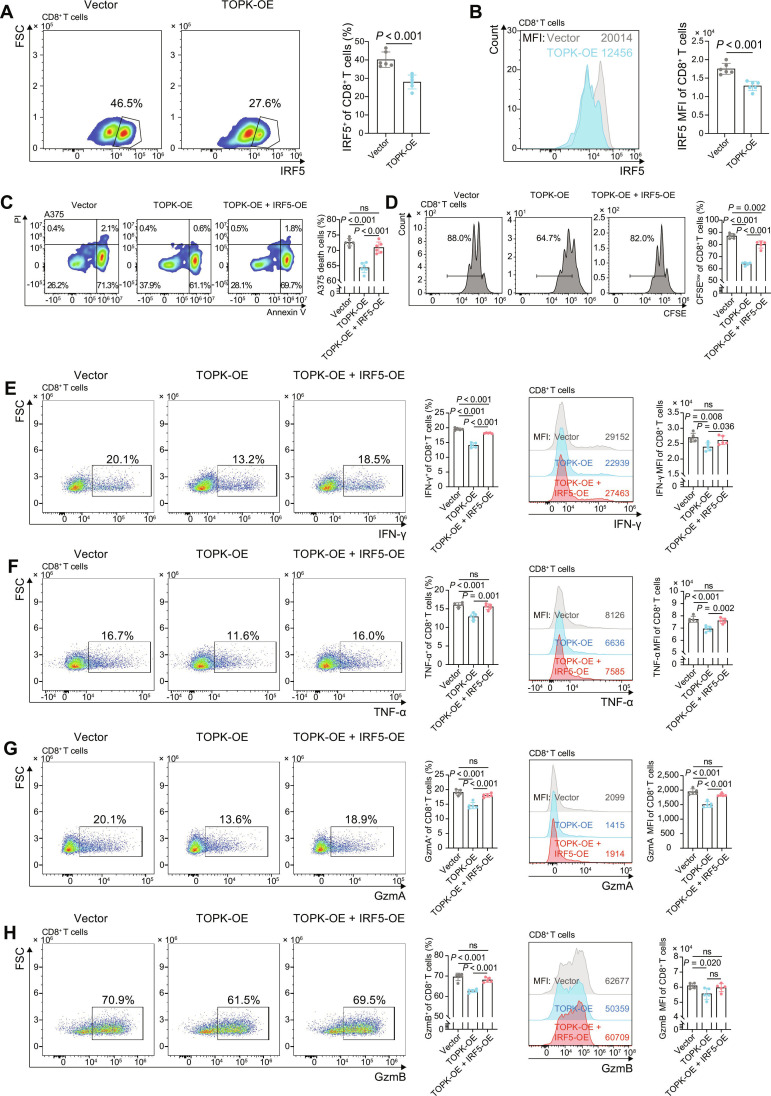
TOPK-mediated suppression of CD8^+^ T cell effector functions through IRF5 inhibition. CD8^+^ T cells isolated from healthy donor PBMCs were activated using anti-CD3/CD28 Dynabeads and transduced with vector (control), TOPK-OE, or TOPK-OE + IRF5-OE5, and cocultured with A375 melanoma cells at an effector-to-target ratio of 10:1 for 48 h (*n* = 6 per group). Representative flow cytometry plots (left) and quantitative analyses (right) of the frequency of IRF5^+^ subsets (A) and IRF5 MFI (B) in CD8^+^ T cells in each group. (C) Representative flow cytometry plots (left) and quantitative analysis (right) of CD8^+^ T cell-mediated A375 cell death in each group, assessed by Annexin V/PI staining. (D) Representative CFSE dilution histograms and quantification of proliferating CD8^+^ T cells in each group. Frequency (left) and MFI (right) of IFN-γ (E), TNF-α (F), GzmA (G), and GzmB (H) in CD8^+^ T cells across each group, shown as representative flow plots/histograms and quantification. Data represent the mean ± SEM (A to H). Unpaired 2-tailed *t* test (A to H). ns: not significant. PBMC, peripheral blood mononuclear cell; TOPK, T-LAK cell-originated protein kinase; IRF5, interferon regulatory factor 5; IFN-γ, interferon-γ; TNF-α, tumor necrosis factor-α; GzmA/B, granzyme A/B; MFI, mean fluorescence intensity; SEM, standard error of the mean; PI, propidium Iodide; CFSE, carboxyfluorescein succinimidyl ester; OE, overexpression.

To validate the clinical relevance of the TOPK–IRF5 axis in human CD8^+^ T cells, we performed in vitro functional assays using primary CD8^+^ T cells isolated from healthy donor peripheral blood. We noted that lentiviral-mediated TOPK overexpression (TOPK-OE) markedly down-regulated IRF5 protein levels compared with those in empty-vector (Vector)-transduced CD8^+^ T cells (Fig. 6A and B). To assess the functional impact of TOPK-OE on human CD8^+^ T cells, we performed cytotoxic T lymphocyte assays. ​Consistently, TOPK-OE CD8^+^ T cells showed reduced cytolytic activity against human melanoma A375 cells and impaired proliferation relative to those in the TOPK-OE (Fig. 6C and D). Additionally, TOPK-OE CD8^+^ T cells produced reduced levels of effector cytokines IFN-γ and TNF-α, as well as cytolytic granules GzmA and GzmB, compared with Vector cells, while perforin levels remained unaltered (Fig. 6E to H and Fig. S14). Importantly, simultaneous IRF5-OE in the context of TOPK-OE rescued these functional deficits (Fig. 6C to H), further supporting the role of IRF5 as a key downstream mediator of the effects of TOPK on CD8^+^ T cell function.

### Targeting TOPK enhances CD8^+^ T cell antitumor effect and synergizes with PD-1 blockade for enhanced tumor suppression

**Fig. 7.**
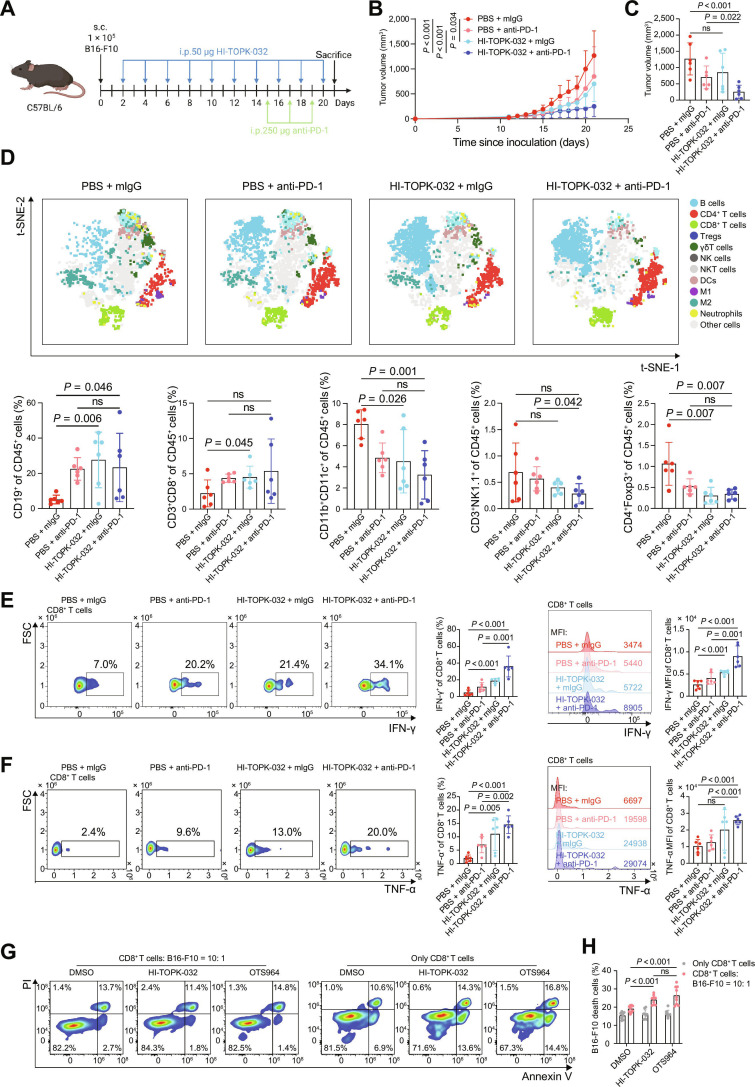
Synergistic antitumor efficacy of combined TOPK inhibition and anti-PD-1 therapy in CD8^+^ T cell-mediated immunity. (A to F) C57BL/6 mice bearing B16-F10 tumors (*n* = 6 per group) were treated with PBS + mIgG, PBS + anti-PD-1, HI-TOPK-032​+ mIgG, or HI-TOPK-032 + anti-PD-1. Subcutaneous B16-F10 tumors were established in C57BL/6 mice, followed by i.p. HI-TOPK-032 dosing every other day from day 3 and i.p. anti-PD-1 (or mIgG) injections on days 15, 17, and 19, with tumor harvest on day 21 for measurement. (A). Tumor growth kinetics and (B) tumor volumes on day 21 post-inoculation (C) in each group. t-SNE projection (top) and frequencies (bottom) of CD19^+^ B cells, CD3^+^CD8^+^ T cells, CD11b^+^CD11c^+^ DCs, CD3^+^NK1.1^+^ NKT cells, and CD4^+^Foxp3^+^ Tregs of CD45^+^ TILs across groups, based on flow cytometry data (D). Frequency (left) and MFI (right) of IFN-γ (E) and TNF-α (F) in CD8^+^ T cells across each group, shown as representative flow plots/histograms and quantification. (G and H) CD8^+^ T cells isolated from WT mice were activated using anti-CD3/CD28 Dynabeads and cocultured with B16-F10 melanoma cells at an effector-to-target ratio of 10:1 for 48 h (*n* = 9 per group), treated with dimethyl sulfoxide (DMSO), HI-TOPK-032, or OTS964. ​Flow cytometry gating (G) and statistical analysis (H) of the effects of HI-TOPK-032 and OTS964 on in vitro cytotoxic activity of CD8^+^ T cells. Data represent the mean ± SEM (B to F and H). Mixed-effects model with group and time as fixed effects and individual mice as random effects (B), unpaired 2-tailed *t* test (C to F and H). ns: not significant. HI-TOPK-032, TOPK-specific inhibitor; IFN-γ, interferon-γ; MFI, mean fluorescence intensity; mIgG, mouse immunoglobulin G; OTS964, small-molecule TOPK inhibitor; PBS, phosphate-buffered saline; SEM, standard error of the mean; TIL, tumor-infiltrating lymphocyte; TNF-α, tumor necrosis factor-α; WT, wild type; s.c., subcutaneous injection; i.p., intraperitoneal injection; t-SNE, t-distributed stochastic neighbor embedding; PI, propidium iodide.

Finally, we evaluated a drug-based TOPK inhibition strategy by treating tumor-bearing mice with the TOPK inhibitor HI-TOPK-032 in a murine tumor model to assess its therapeutic potential as an immunotherapy target. Tumor growth was significantly reduced in mice treated with HI-TOPK-032 + mouse immunoglobulin G (mIgG) compared with the PBS + mIgG control group. Treatment with PBS + anti-PD-1 also significantly restrained tumor growth relative to PBS + mIgG. Notably, the HI-TOPK-032 + anti-PD-1 combination produced the greatest tumor suppression among all 4 groups (PBS + mIgG, PBS + anti-PD-1, HI-TOPK-032 + mIgG, and HI-TOPK-032 + anti-PD-1) (Fig. 7A to C and Fig. S15A). Based on the potential off-target effects of inhibitors, we further validated these findings in *Cd8*^Cre^*Topk*^fl/fl^ mice treated with anti-PD-1, and noted the consistent results (Fig. S15B to D).

Additionally, flow cytometric analysis showed that compared with the PBS + mIgG control group, the HI-TOPK-032 + anti-PD-1 combination increased the proportions of CD19^+^ cells in tumors while decreasing the proportions of CD11c^+^ cells and CD4^+^Foxp3^+^ cells. Moreover, IFN-γ and TNF-α secretion by tumor-infiltrating CD8^+^ T cells was elevated in the combination group relative to PBS + mIgG (Fig. 7D to F). In contrast, the proportions of other immune cell subsets, including CD3^+^CD4^+^ cells, CD86^+^ cells, CD206^+^ cells, and CD3^−^NK1.1^+^ cells, were not significantly changed between these 2 groups (Fig. S15E). Likewise, no significant differences were observed in the proportions of major immune cell subsets in the LNs, PBMCs, or SP between these 2 groups (Fig. S15F to H). However, no significant changes were observed in the perforin, GzmA, or GzmB levels (Fig. S16). Furthermore, pharmacological inhibition of TOPK (HI-TOPK-032 and OTS964) increased the CD8^+^ T cell-mediated cytotoxic responses against melanoma B16-F10 cells in vitro (Fig. 7G and H). Overall, these findings underscore the potential therapeutic value of targeting TOPK for enhanced antitumor immunity.

## Discussion

This study shows that TOPK is expressed by a subset of tumor-infiltrating CD8^+^ T cells and that genetic loss of *Topk* in CD8^+^ T cells is associated with stronger antitumor activity in melanoma, as evidenced by enhanced effector cytokines (IFN-γ and TNF-α) and GzmB, transcriptional and phospho-signatures consistent with heightened activation, and improved control in adoptive transfer and inhibitor-combination settings. Mechanistically, our data demonstrate that TOPK restrains IRF5 expression in T cells; in human primary CD8^+^ cells, TOPK-OE impairs cytotoxic readouts and cytokine production, whereas coexpression of IRF5 mitigates these effects, being consistent with an IRF5-linked regulatory axis. Our pharmacologic observations are also consistent with prior work showing that TOPK inhibition enhances CAR-T efficacy [44]. Together, these results support a model in which TOPK limits CD8^+^ T cell effector programming within the TME.

TOPK is highly expressed in proliferative tissues (e.g., testes and bone marrow) and is overexpressed in multiple cancers, including colorectal [68,69], breast [70], and lung cancers [71]; it drives tumorigenesis by enhancing cancer cell cycle progression, DNA damage response, and apoptosis resistance. TOPK acts as an oncogenic kinase driving tumor proliferation and metastasis by activating pathways like ERK/STAT3 or regulating transcription factors such as NF-κB [72–75]. For example, TOPK enhances tumorigenic properties in colorectal cancer via a reciprocal feedback loop with ERK2, mediating mutual phosphorylation and ERK2 downstream signaling activation [72]. Moreover, TOPK promotes the malignant progression of cutaneous squamous cell carcinoma by up-regulating HDAC1 to activate the NF-κB pathway and promote autophagy [73], then mediating TGF-β1-induced epithelial–mesenchymal transition and invasion in breast cancer cells via NF-κB/Snail signaling [74]. Furthermore, it up-regulates inducible nitric oxide synthase (iNOS) gene expression in T cell leukemia Jurkat cells or macrophage leukemic RAW 264.7 cells via NF-κB activation in response to lipopolysaccharide [75]. Previous studies have demonstrated that TOPK promotes tumor cell proliferation [37,40]; our data highlight that TOPK also suppresses CD8^+^ T cell-mediated antitumor immunity within the TME, thereby contributing to tumor progression from both tumor-intrinsic and immune-regulatory aspects.

Mechanistically, transcriptional profiling of TOPK-KO CD8^+^ T cells revealed that a key transcription factor, IRF5, was up-regulated compared with that in WT cells. IRF5 drives effector gene transcription in CD4^+^ T cells and pro-inflammatory macrophage polarization [76–78]. In CD8^+^ T cells, IRF5 expression ​curtails functional exhaustion​ during chronic viral infections [62]. Notably, silencing IRF5 in CD8^+^ T cells ​augments IFN-γ transcriptional expression​ in rheumatoid arthritis [79]. Our data indicate that genetic TOPK deficiency enhances IRF5 expression, which may restore CD8^+^ T cell effector function by transcriptionally activating IRF5-mediated effector pathways. Furthermore, in human primary CD8^+^ T cells, TOPK-OE reduces cytokine secretion, whereas co-overexpression of TOPK and IRF5 abolishes TOPK-mediated suppression of effector function. These results, together with prior studies implicating IRF5 in memory T cell maintenance and pro-inflammatory polarization [80,81], suggest a potential link between TOPK activity and IRF5-associated programs in CD8^+^ T cells. However, the molecular basis of this relationship remains to be defined, as we did not detect a direct TOPK–IRF5 interaction by Co-IP and have not yet functionally validated the upstream/downstream regulatory pathway. Therefore, the proposed model should be considered indirect and hypothesis-generating. Additionally, our phospho-protein and transcriptional profiling findings indicate that TOPK limits key activation signaling pathways including LCK and ZAP-70, reinforcing its role as a brake for TCR-driven activation.

Furthermore, pharmacological TOPK inhibition—using either HI-TOPK-032 or OTS964 [82]—enhanced CD8^+^ T cell-mediated tumor cell killing. Notably, combined HI-TOPK-032 and anti-PD-1 treatment demonstrated significantly greater antitumor efficacy than did either monotherapy. These findings underscore the clinical translational potential of TOPK targeting in both cellular therapies (e.g., adoptive cell transfer) and small-molecule inhibitor-based regimens. Although the changes in specific immune subsets were modest, we noted a reduction in tumor-infiltrating Tregs under combination therapy, which may relieve suppressive pressure within the TME and contribute to enhanced antitumor immunity. Although this effect did not emerge as a central mechanism in our study, it supports the concept that TOPK targeting affects multiple components of the T cell regulatory network.

Despite the strength of these findings, several limitations warrant consideration. First, while in vitro experiments with human T cells support the functional conservation of TOPK-mediated suppression, additional work using humanized tumor models is required to verify therapeutic translatability. Second, the pharmacological inhibitors used may exert off-target effects on tumor cells or other immune populations, necessitating improved specificity and pharmacokinetic evaluation. Third, the upstream triggers of TOPK-OE in tumor-infiltrating CD8^+^ T cells—whether tumor-derived metabolites (e.g., lactate and kynurenine) or persistent TCR stimulation—require further elucidation. Additionally, TOPK deletion resulted in broader immune alterations in germline knockout mice, indicating possible roles beyond CD8^+^ T cells that were not completely explored here.

Together, our data support a model in which TOPK functions as a regulatory brake on the activation and antitumor responses of CD8^+^ T cells within the melanoma microenvironment. Relieving this suppression, either genetically or pharmacologically, may potentiate T cell-mediated tumor control and improve the efficacy of PD-1 checkpoint blockade. Further investigation is thus essential to refine TOPK-targeted approaches for clinical application.

## Conclusions

This study reveals that TOPK is expressed in tumor-infiltrating CD8^+^ T cells, where it promotes their dysfunction by suppressing the expression of the transcription factor IRF5. By integrating mechanistic insights with therapeutic validation, we demonstrate that combined targeting of TOPK/PD-1 restores the antitumor activity of tumor-infiltrating CD8^+^ T cells, establishing TOPK inhibition as a promising therapeutic approach for patients with melanoma and other cancers.

## Ethical Approval

This study was approved by the Medical Ethical Committee of Guangdong Medical University Affiliated the First Dongguan Hospital (approval no. YJYS202402018). Frozen CD8^+^ T cells from healthy donor were obtained from ORIBIOTECH Co. Ltd., and ethical approval for their use was obtained from the Ethics Committee of Shanghai Zhaxin combination of Chinese traditional and western medicine hospital (approval no. 202409). Human melanoma tumor tissue sections were commercially purchased from Changsha Yaxiang Biotechnology Co. Ltd., and ethical approval for their use was obtained from the Ethics Committee of Shanghai Kaiwang Biotechnology Co. Ltd. (approval no. KW250618). Informed consent was obtained from all participants prior to their inclusion in the study. All animal experiments were conducted in accordance with institutional guidelines and approved by the Animal Ethics Committee of Guangdong Medical University (approval no. GDY2402030).

## Data Availability

The results of this study integrate 2 complementary data sources: (a) publicly available scRNA-seq datasets from the GEO (https://www.ncbi.nlm.nih.gov/geo/) and the ArrayExpress database at EMBL-EBI (www.ebi.ac.uk/arrayexpress), including melanoma tumor tissues (GSE215120) and normal LN specimens from deceased donors (GSE193449 and E-MTAB-11536 ), and (b) newly generated experimental datasets deposited in the National Genomics Data Center (https://ngdc.cncb.ac.cn/) under accession number PRJCA039759 and PRJCA041659, which encompass bulk RNA-seq profiles of CD8^+^ T cells isolated from *Topk*^−/−^ and WT mice under resting/activated conditions (subSAM140970), 10x scRNA-seq data of tumor-infiltrating immune cells from *Cd8*^Cre^*Topk*^fl/fl^ and *Topk*^fl/fl^ mice (subSAM143096), and bulk RNA-seq data of TOPK-KO/WT Jurkat cell lines (subSAM143405). Biological materials are available on request from the corresponding author.
