# Supplementary material for: TOPK Suppresses the CD8+ T Cell Antitumor Immunity via Modulation of IRF5 Expression
Source: Cancer Commun (Lond). 2026 Mar 20;46:0021. doi: 10.34133/cancomm.0021 (PMC13003159; doi:10.34133/cancomm.0021)
Supplement: Supplementary 1 — Figs. S1 to S16 Tables S1 and S2 [file cancomm.0021.f1.zip › 00173-Supplementary Materials-final.docx]

**Supplementary Materials**

**TOPK Suppresses the CD8^+^ T Cell Antitumor Immunity via Modulation of IRF5 Expression**

Nianke Zang^1,†^, Jinfeng Gan^2,3,†^, Ye Chen^4,†^, Zheng Huang^1,2^, Chichu Xie^2,3^, Junlong Dang^4^, Changyuan Huang^1^, Linjie Yang^1^, Xuelian Chen^1^, Guangli Rong^1,5^, Jianbo Sun^1,6^, Yiming Shao^1,7^, Julie Wang^1,4^, Guangying Qi^2,3,*^, Yu Liu^1,2,8,*^, Song Guo Zheng^1,4,9,*^

^1^Clinical Research Center, The First Dongguan Affiliated Hospital, Guangdong Medical University, Dongguan, Guangdong, P. R. China;

^2^Guangxi Key Laboratory of Tumor Immunology and Microenvironmental Regulation, Guilin Medical University, Guilin, Guangxi, P. R. China;

^3^Guangxi Health Commission Key Laboratory of Tumor Immunology and Receptor‑Targeted Drug Basic Research, Guilin Medical University, Guilin, Guangxi, P. R. China;

^4^Department of Immunology, School of Cell and Gene Therapy, Songjiang Institute and Songjiang Hospital Affiliated to the Shanghai Jiao Tong University School of Medicine, Shanghai, P. R. China;

^5^State Key Laboratory of Pathogenesis, Prevention and Treatment of High Incidence Diseases in Central Asia, The First Dongguan Affiliated Hospital, Guangdong Medical University, Dongguan, Guangdong, P. R. China;

^6^Dongguan Key Laboratory of Chronic Inflammatory Diseases, The First Dongguan Affiliated Hospital, Guangdong Medical University, Dongguan, Guangdong, P. R. China;

^7^The Key Laboratory of Sepsis Translational Medicine, Guangdong Medical University; Dongguan Key Laboratory of Sepsis Translational Medicine; The First Dongguan Affiliated Hospital, Guangdong Medical University, Dongguan, Guangdong, P. R. China;

^8^Guangdong Provincial Key Laboratory of Medical Immunology and Molecular Diagnostics, School of Medical Technology, Guangdong Medical University, Dongguan, Guangdong, P. R. China;

^9^State Key Laboratory of Innovative Immunotherapy, Shanghai Jiao Tong University, Shanghai, P. R. China.

^†^Nianke Zang, Jinfeng Gan, and Ye Chen contributed equally to this work.

^*^Corresponding authors:

SGZ (Song Guo Zheng), [Song.Zheng@shsmu.edu.cn](mailto:Song.Zheng@shsmu.edu.cn);

YL (Yu Liu), [liuyu177@gdmu.edu.cn](mailto:liuyu177@gdmu.edu.cn);

GQ (Guangying Qi), [qgy@glmc.edu.cn](mailto:qgy@glmc.edu.cn).


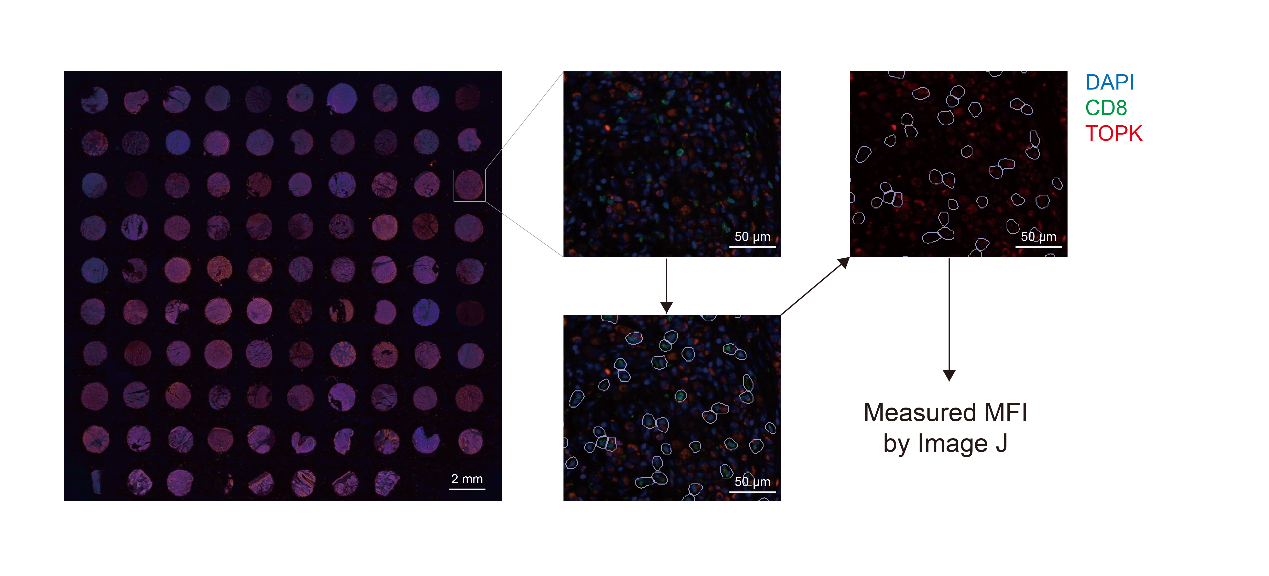


**Supplementary Figure S1. Multiplex immunofluorescence analysis of melanoma tissue microarrays and data processing workflow.**

Multiplex fluorescence analysis of TOPK expression in CD8⁺ T cells across pathological grades of melanoma (*n* = 90 patients). Nuclei were counterstained with DAPI (blue), CD8⁺ T cells were labeled with an anti-CD8 antibody (green), and TOPK was detected with an anti-TOPK antibody (red). CD8⁺ T cell regions were segmented using Cellpose (v2.0) cyto2 model (diameter parameter auto-optimized based on tissue morphology), followed by manual verification of the masks for accuracy. The verified CD8⁺ masks were then applied to the TOPK channel, and TOPK fluorescence within CD8⁺ regions were quantified in ImageJ (v1.53e) using integrated density; MFI was calculated by normalizing integrated density to mask area.

Abbreviations: TOPK, T-LAK cell-originated protein kinase; MFI, mean fluorescence intensity; DAPI, 4’,6-diamidino-2-phenylindole.


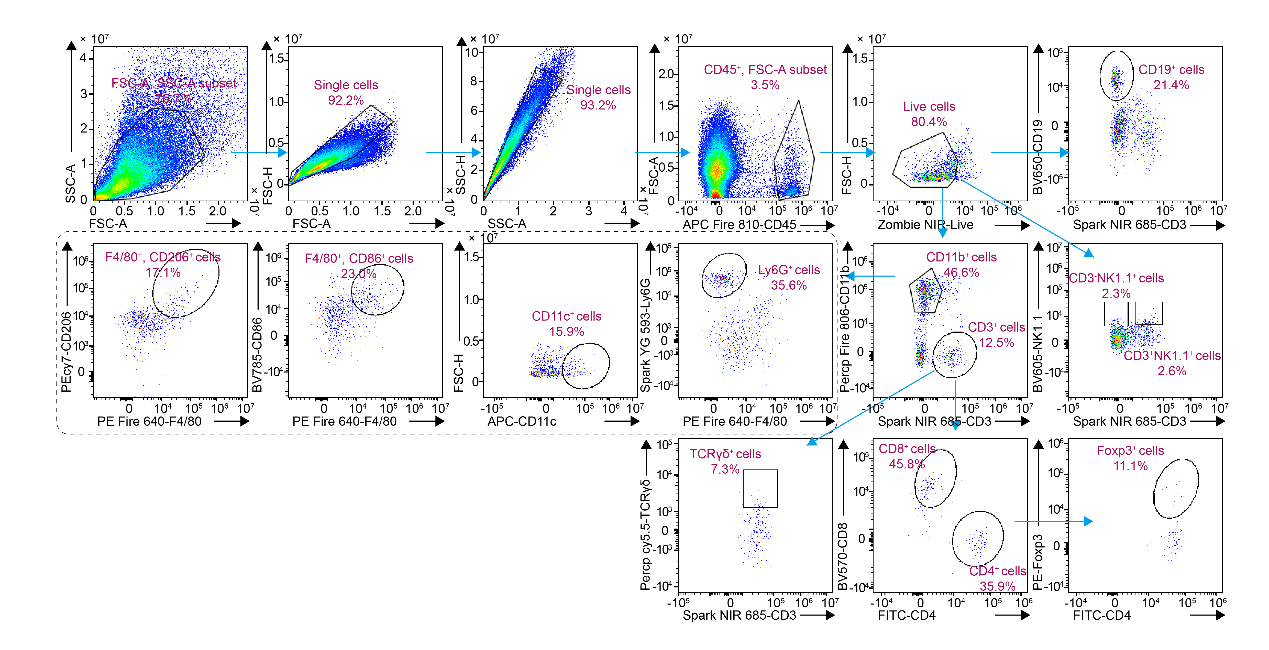


**Supplementary Figure S2. Gating strategy for flow cytometry data analysis.**

The gating strategy for flow cytometry data analysis involved a panel of fluorescently conjugated antibodies to delineate tumor-infiltrating immune subsets, including: B cells (CD19^+^CD3^-^), CD4^+^ T cells (CD3^+^CD4^+^CD8^-^), CD8^+^ T cells (CD3^+^CD8^+^CD4^-^), Tregs (CD4^+^Foxp3^+^), γδ T cells (TCRγδ^+^CD3^+^), NK cells (NK1.1^+^CD3^−^), NKT cells (NK1.1^+^CD3^+^), M1 macrophages (CD11b^+^F4/80^high^CD86^+^), and M2 macrophages (CD11b^+^F4/80^high^CD206^+^), DCs (CD11b^+^CD11c^+^).

Abbreviations: B cell, B lymphocyte; Treg, regulatory T cell; γδ T cell, gamma delta T cell; NK, natural killer cell; NKT, natural killer T cell; M1, classically activated macrophage; M2, alternatively activated macrophage; DC, dendritic cell.


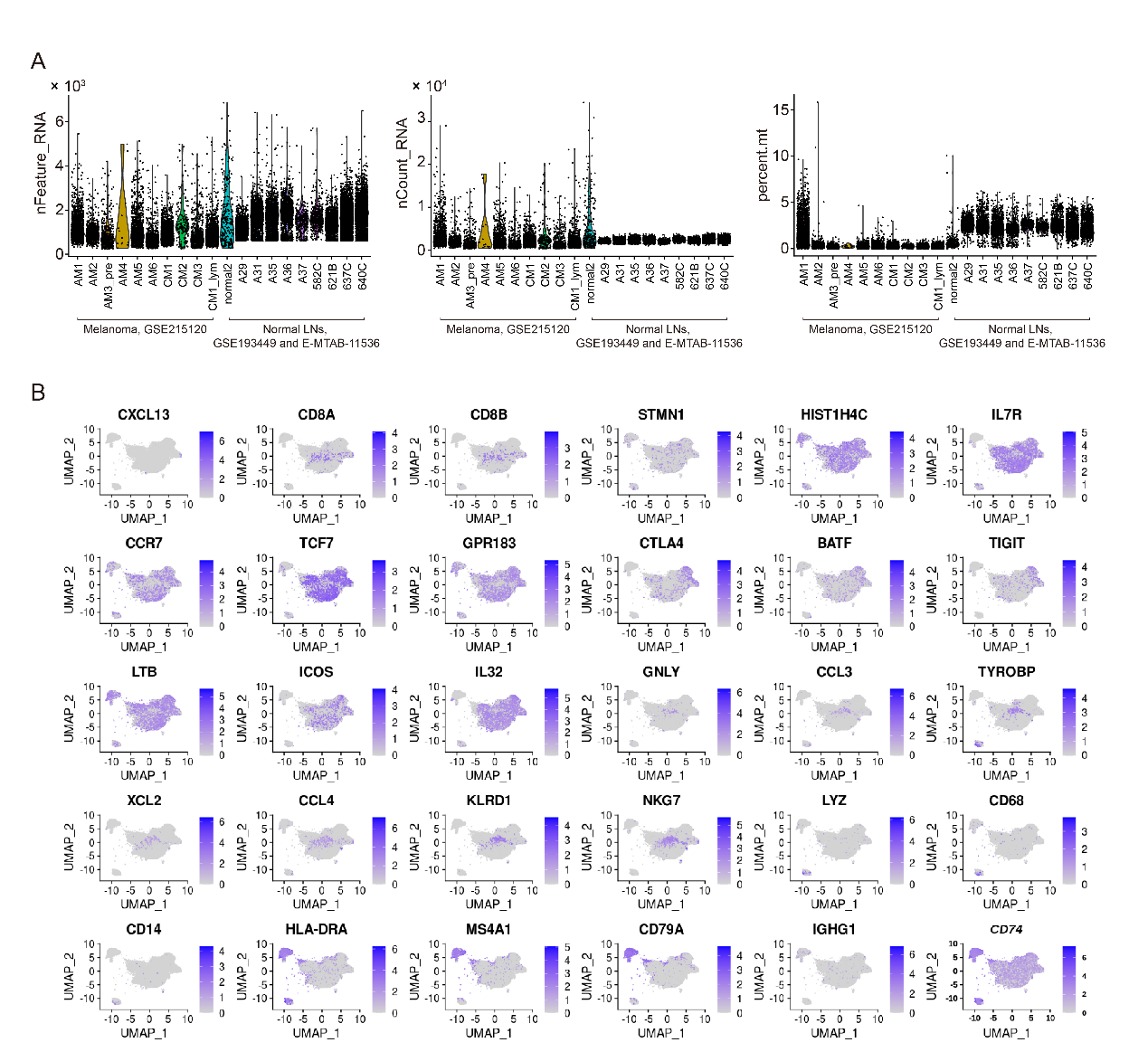


**Supplementary Figure S3. scRNA-seq quality control and lymphocytes annotation in human melanoma tissues.**

scRNA-seq analysis of lymphocytes from 10 untreated melanoma patients (16,011 cells post-quality control) and 10 LNs (23,449 cells post-quality control) was performed. The melanoma samples were obtained from GSE215120, with the following sample identifiers: AM1, AM2, AM3_pre, AM4, AM5, AM6, CM1, CM2, CM3, CM1_lym. The LN samples were obtained from GSE193449 and E-MTAB-11536, with normal2 from GSE193449 and A29, A31, A35, A36, A37, 582C, 621B, 637C, 640C from E-MTAB-11536.

(A) scRNA-seq quality metrics across donor samples, including nFeature_RNA, nCount_RNA, percent.mt (mitochondrial gene content).

(B) Cell type classification of lymphocytes was performed based on canonical lineage-specific markers using the integrated scRNA-seq dataset compiled from GSE215120, GSE193449, and E-MTAB-11536, and marker expression patterns were visualized using FeaturePlot.

Abbreviations: scRNA-seq, single-cell RNA sequencing; LN, lymph node; UMAP, Uniform Manifold Approximation and Projection; CXCL13, C-X-C motif chemokine ligand 13; CD8A, CD8a molecule; CD8B, CD8b molecule; STMN1, stathmin 1; HIST1H4C, histone cluster 1 H4 family member C; IL7R, interleukin 7 receptor; CCR7, C-C motif chemokine receptor 7; TCF7, transcription factor 7; GPR183, G protein-coupled receptor 183; CTLA4, cytotoxic T-lymphocyte associated protein 4; BATF, basic leucine zipper ATF-like transcription factor; TIGIT, T cell immunoreceptor with Ig and ITIM domains; LTB, lymphotoxin beta; ICOS, inducible T cell costimulatory; IL32, interleukin 32; GNLY, granulysin; CCL3, C-C motif chemokine ligand 3; TYROBP, TYRO protein tyrosine kinase binding protein; XCL2, X-C motif chemokine ligand 2; CCL4, C-C motif chemokine ligand 4; KLRD1, killer cell lectin like receptor D1; NKG7, natural killer cell granule protein 7; LYZ, lysozyme; CD68, cluster of differentiation 68; CD14, cluster of differentiation 14; HLA-DRA, major histocompatibility complex, class II, DR alpha; MS4A1, membrane spanning 4-domains A1; CD79A, CD79a molecule; IGHG1, immunoglobulin heavy constant gamma 1; CD74, cluster of differentiation 74.


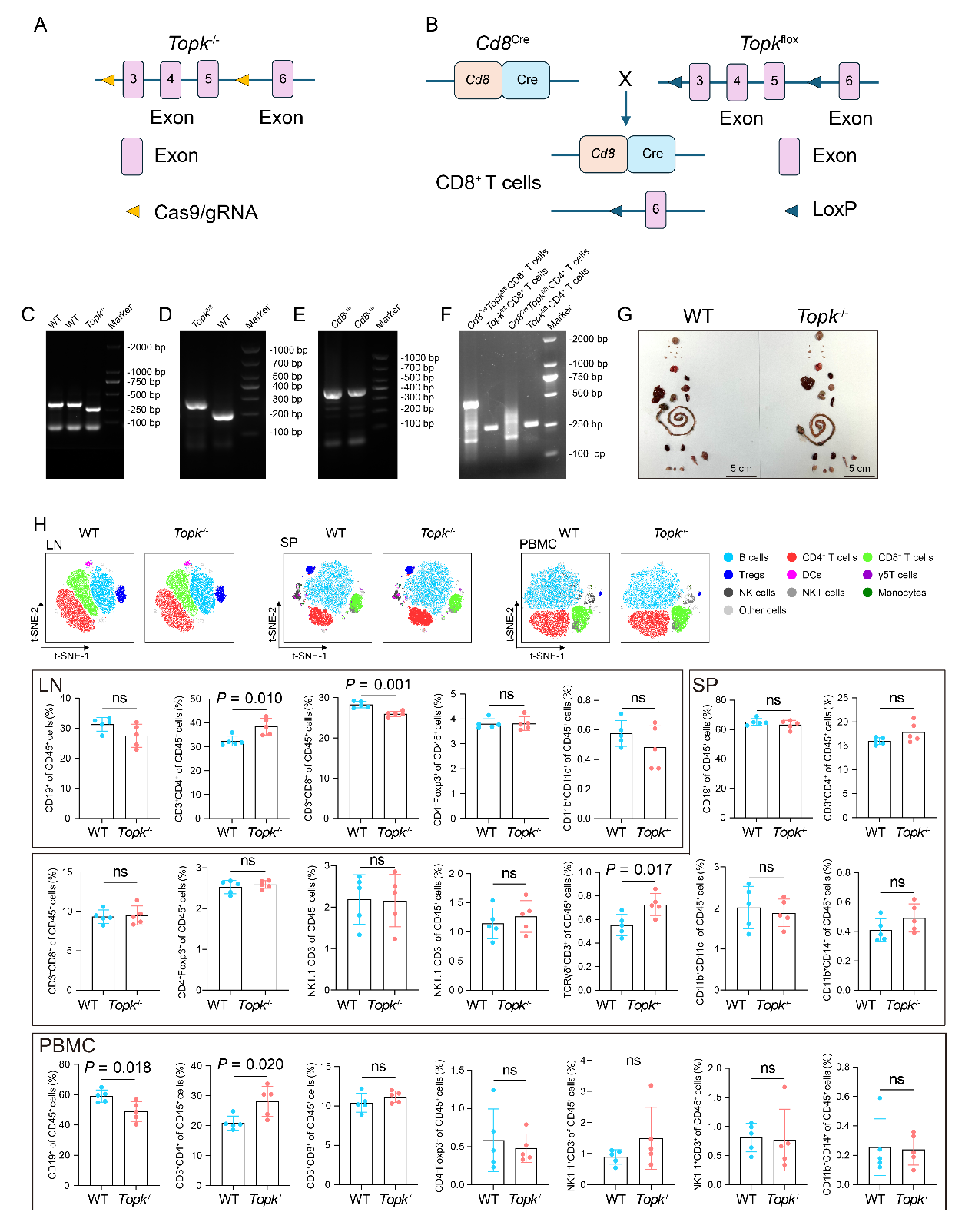


**Supplementary Figure S4. Validation of systemic and CD8^+^ T cell-specific *Topk* knockout mice.**

(A) Schematic of the *Topk* whole-genome knockout (KO) strategy.

(B) Schematic of the CD8^+^ T cell-specific *Topk* knockout strategy.

(C-F) PCR and agarose gel electrophoresis analyses: Genotyping of *Topk*^-/-^ mice, validation of *Topk* genomic fragment deletion (C); Genotyping of *Topk*^fl/fl^ mice, verification of loxP site insertion flanking the *Topk* exons (D); Genotyping of *Cd8^Cre^* mice, detection of Cre recombinase transgene insertion (E); Genotyping of *Cd8^Cre^Topk*^fl/fl^ mice, confirmation of *Topk* fragment deletion in CD8^+^ T cell genomic DNA (F).

(G) Photograph of major organs (liver, spleen and kidney) from WT and *Topk*^-/-^ mice.

(H) t-SNE visualization and quantification of immune cell proportions in the LN, SP, and PBMCs of WT and *Topk*^-/-^ mice (*n* = 5 per group).

Data are shown as the mean ± SEM (H). Unpaired two-tailed *t*-tests (H). ns, not significant.

Abbreviations: KO, knockout; PCR, polymerase chain reaction; SP, spleen; PBMC, peripheral blood mononuclear cell; LN, lymph node; WT, wild type; t-SNE, t-distributed stochastic neighbor embedding; SEM, standard error of the mean; Treg, regulatory T cell; γδT cell, gamma delta T cell; NK cell, natural killer cell; NKT cell, natural killer T cell; DC, dendritic cell; Cre, Cyclization recombinase; loxP, Locus of crossover (x) in P1; Cas9, CRISPR-associated protein 9.


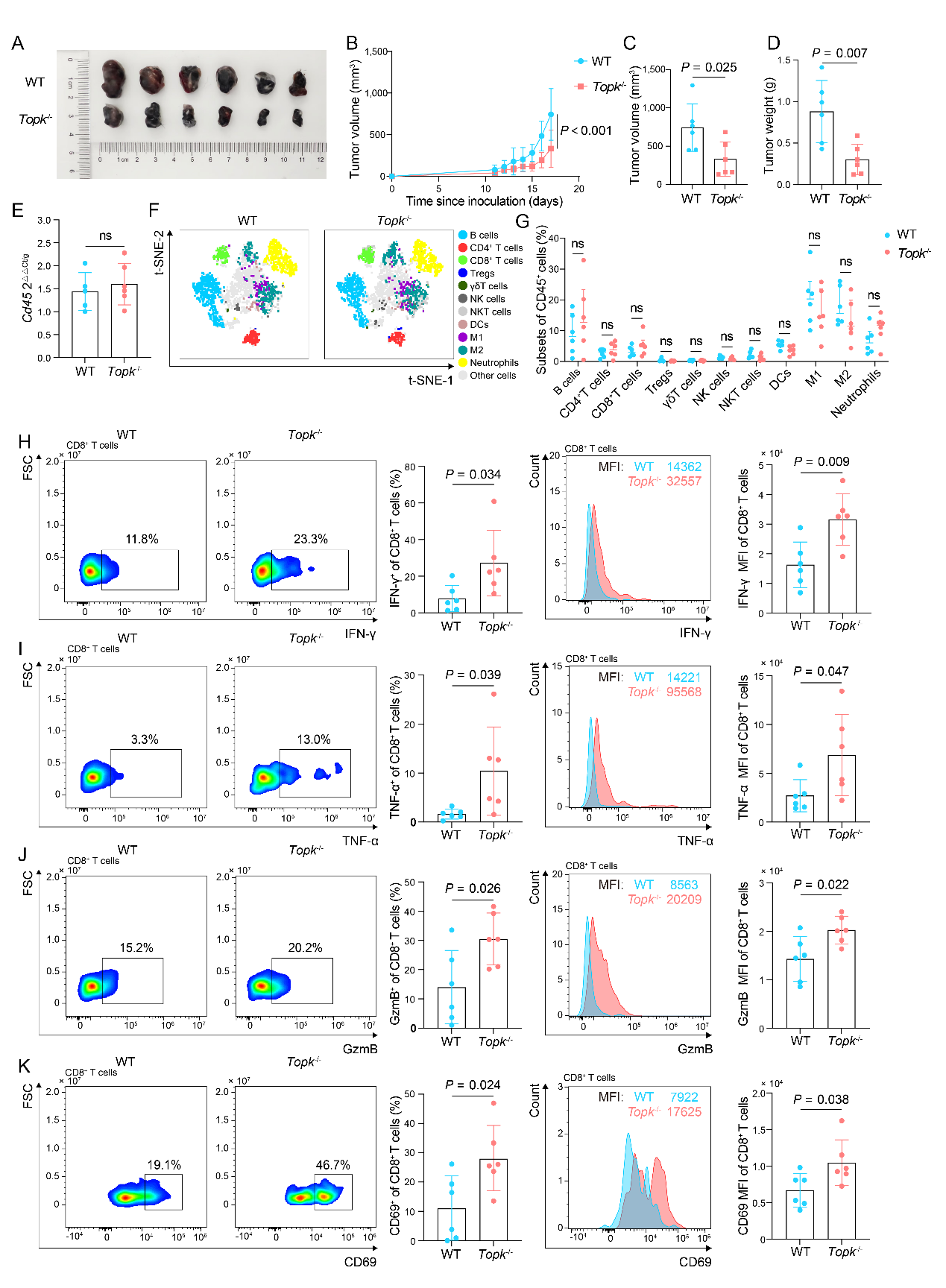


**Supplementary Figure S5. Impact of *Topk* deletion in mice on antitumor immunity.**

*Topk*^-/-^ mice and WT littermate controls (*n* = 6 per group) were inoculated subcutaneously with 0.5 × 10⁶ B16-F10 cells.

(A-D) Photographs of the tumor collected at day 17 since inoculation (A), tumor growth kinetics (B), tumor volumes at day 17 since inoculation (C), and tumor weights at day 17 since inoculation (D) in *Topk*^-/-^ *vs.* WT mice.

(E) Cd45 mRNA expression levels in tumor tissues from WT and *Topk*^-/-^ mice were quantified using qPCR (normalized to *Gapdh*).

(F) t-SNE visualization of TIL subsets using flow cytometry (*Topk*^-/-^ *vs.* WT).

(G) Frequencies of TIL subsets in *Topk*^-/-^ *vs.* WT mice.

(H-K) Frequency (left) and MFI (right) of IFN-γ (H), TNF-α (I), GzmB (J) and CD69 (K) in CD8⁺ T cells across each group shown as representative flow plots/histograms and quantification.

Data represent the mean ± SEM (B-E, G-K). Mixed-effects model with group and time as fixed effects and individual mice as random effects (B), unpaired two-tailed *t*-test (C-E, G-H). ns: not significant.

Abbreviations: WT, wild type; KO, knockout; q-PCR, Quantitative real-time polymerase chain reaction; TIL, Tumor-infiltrating lymphocyte; t-SNE, t-distributed stochastic neighbor embedding; IFN-γ, Interferon-gamma; TNF-α, Tumor necrosis factor α; GzmB, Granzyme B; CD69, cluster of differentiation 69; SEM, Standard error of the mean; *Gapdh*, Glyceraldehyde-3-phosphate dehydrogenase; Treg, regulatory T cell; γδT cell, gamma delta T cell; NK cell, natural killer cell; NKT cell, natural killer T cell; DC, dendritic cell; M1/M2, macrophage type 1/2; Neutrophil, neutrophil granulocyte.


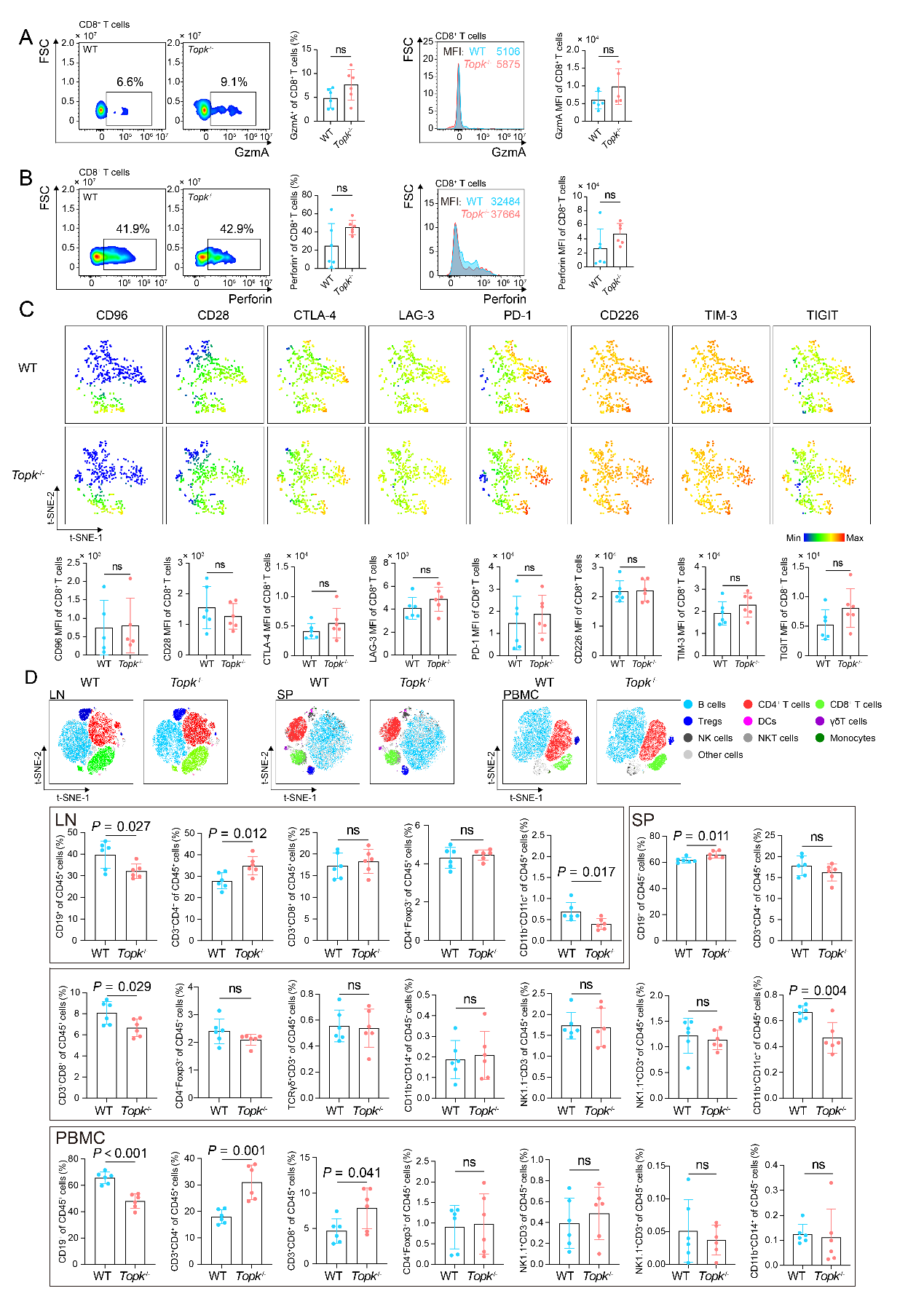


**Supplementary Figure S6. Supplementary analysis of immune cells in WT and *Topk*^-/-^ mice.**

*Topk*^-/-^ mice and WT littermate controls (*n* = 6 per group) were inoculated subcutaneously with 0.5 × 10⁶ B16-F10 cells.

(A-B) Frequency (left) and MFI (right) of GzmA (A) and Perforin (B) in CD8⁺ T cells across groups, shown as representative flow plots/histograms and quantification.

(C) t-SNE visualization and quantification of CD96, CD28, CTLA-4, LAG-3, PD-1, CD226, TIM-3, and TIGIT expression in tumor-infiltrating CD8^+^ T cells from WT *vs.* *Topk*^-/-^ mice.

(D) t-SNE plots and quantification of immune cell proportions in the LN, PBMCs, and SP of tumor-bearing WT and *Topk*^-/-^ mice.

Data are shown as the mean ± SEM (A-D). Unpaired two-tailed *t*-tests (A-D). ns, not significant.

Abbreviations: WT, wild type; GzmA, granzyme A; CTLA-4, cytotoxic T-lymphocyte-associated protein 4; PD-1, programmed cell death protein 1; TIGIT, T cell immunoreceptor with Ig and ITIM domains; TIM-3, T cell immunoglobulin and mucin domain-containing protein 3; LAG-3, lymphocyte activation gene 3; CD226, cluster of differentiation 226; SP, spleen; LN, lymph node; PBMC, peripheral blood mononuclear cell; SEM, standard error of the mean; MFI, mean fluorescence intensity; Treg, regulatory T cell; γδT cell, gamma delta T cell; NK cell, natural killer cell; NKT cell, natural killer T cell; DC, dendritic cell.


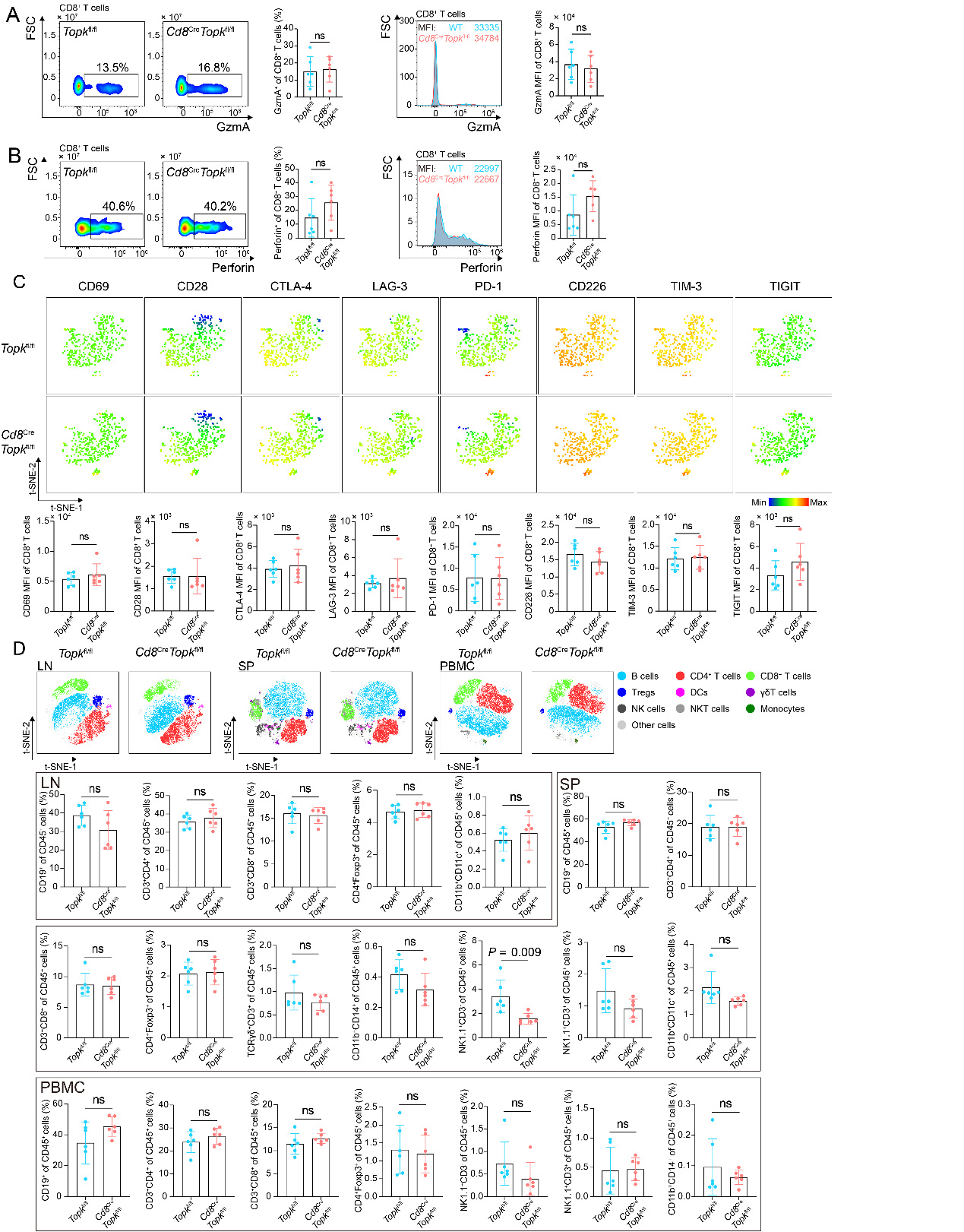


**Supplementary Figure S7. Analysis of immune cells in *Topk*^fl/fl^ and *Cd8*^Cre^*Topk*^fl/fl^ mice.**

*Cd8*^Cre^*Topk*^fl/fl^ mice and *Topk*^fl/fl^ littermate controls (*n* = 6 per group) were subcutaneously inoculated with 0.5 × 10⁶ B16-F10 cells. Data shown represent one of three independent experiments.

(A-B) Frequency (left) and MFI (right) of GzmA (A) and Perforin (B) in CD8⁺ T cells across groups, shown as representative flow plots/histograms and quantification.

(C) t-SNE visualization and quantification of CD69, CD28, CTLA-4, LAG-3, PD-1, CD226, TIM-3, and TIGIT expression in tumor-infiltrating CD8^+^ T cells from *Cd8*^Cre^*Topk*^fl/fl^ and *Topk*^fl/fl^ mice.

(D) t-SNE plots and quantification of immune cell proportions in the LN, PBMCs, and SP of tumor-bearing *Cd8*^Cre^*Topk*^fl/fl^ and *Topk*^fl/fl^ mice.

Data are shown as the mean ± SEM (A-D). Unpaired two-tailed *t*-tests (A-D). ns, not significant.

Abbreviations: *Topk*^fl/fl^, *Topk* floxed mouse; *Cd8*^Cre^*Topk*^fl/fl,^ CD8-specific *Topk* conditional knockout mouse; GzmA, granzyme A; CD69, cluster of differentiation 69; CD28, cluster of differentiation 28; CTLA-4, cytotoxic T-lymphocyte-associated protein 4; PD-1, programmed cell death protein 1; TIGIT, T cell immunoreceptor with Ig and ITIM domains; TIM-3, T cell immunoglobulin and mucin domain-containing protein 3; LAG-3, lymphocyte activation gene 3; CD226, cluster of differentiation 226; SP, spleen; LN, lymph node; PBMC, peripheral blood mononuclear cell; SEM, standard error of the mean; MFI, mean fluorescence intensity; Treg, regulatory T cell; γδT cell, gamma delta T cell; NK cell, natural killer cell; NKT cell, natural killer T cell; DC, dendritic cell.


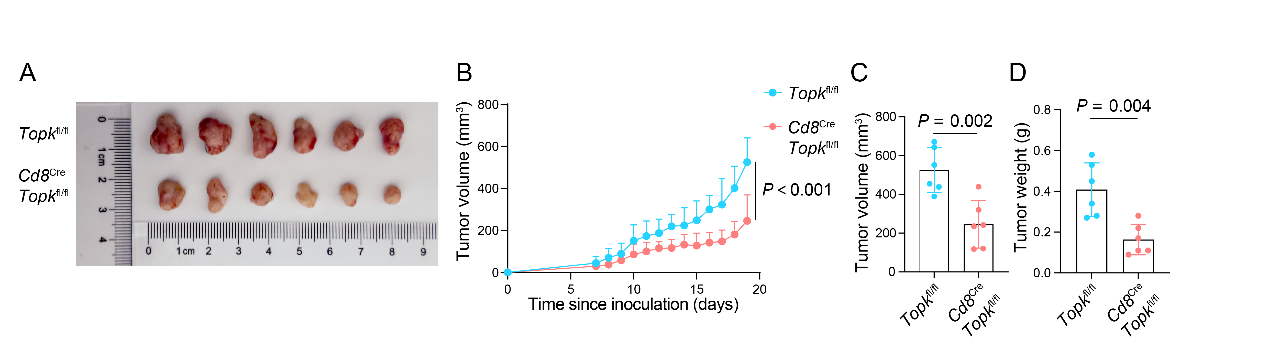


**Supplementary Figure S8. TOPK deficiency enhances antitumor immunity in MC38 colorectal tumor model.**

*Cd8*^Cre^*Topk*^fl/fl^ mice and *Topk*^fl/fl^ littermate controls (*n* = 6 per group) were inoculated subcutaneously with 0.5 × 10⁶ MC38 cells. Photographs of the tumor collected at day 19 since inoculation (A), tumor growth kinetics (B), tumor volumes at day 19 since inoculation (C), tumor weights at day 19 since inoculation (D) in *Cd8*^Cre^*Topk*^fl/fl^ mice and *Topk*^fl/fl^ mice.

Data are shown as the mean ± SEM. Mixed-effects model, group and time as fixed effects and individual mice as random effects (B), unpaired two-tailed *t*-test (C-D).

Abbreviations: *Topk*^fl/fl^, *Topk* floxed mouse; *Cd8*^Cre^*Topk*^fl/fl,^ CD8-specific *Topk* conditional knockout mouse; SEM, Standard error of the mean.


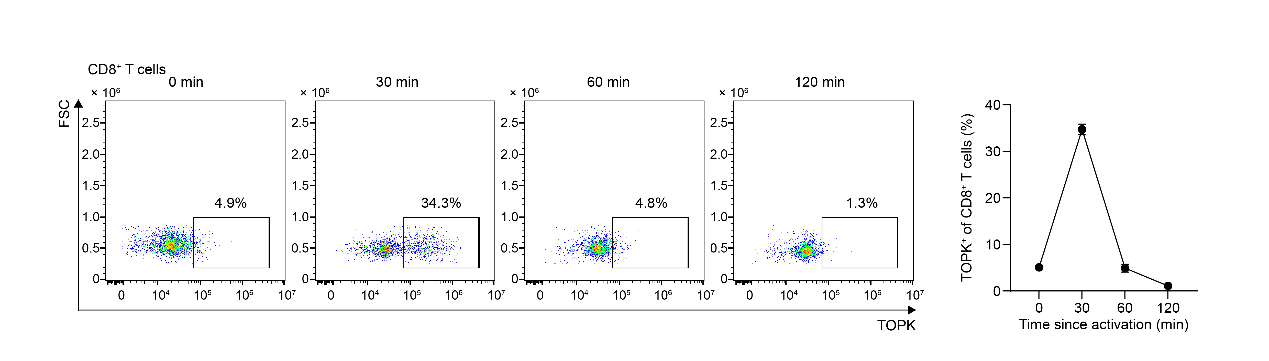


**Supplementary Figure S9. TOPK expression in CD8^+^ T cells during activation.**

CD8⁺ T cells isolated from WT mice were stimulated with anti-CD3/CD28 Dynabeads. The data shown represent one of three independent experiments. ​Representative flow cytometry plots (left) and quantitative analysis (right) of the frequency of TOPK⁺ cells among CD8⁺ T cells at 0, 30, 60, and 120 min post activation.

Abbreviations: WT, wild type; TOPK, T-LAK cell-originated protein kinase; min, minute.


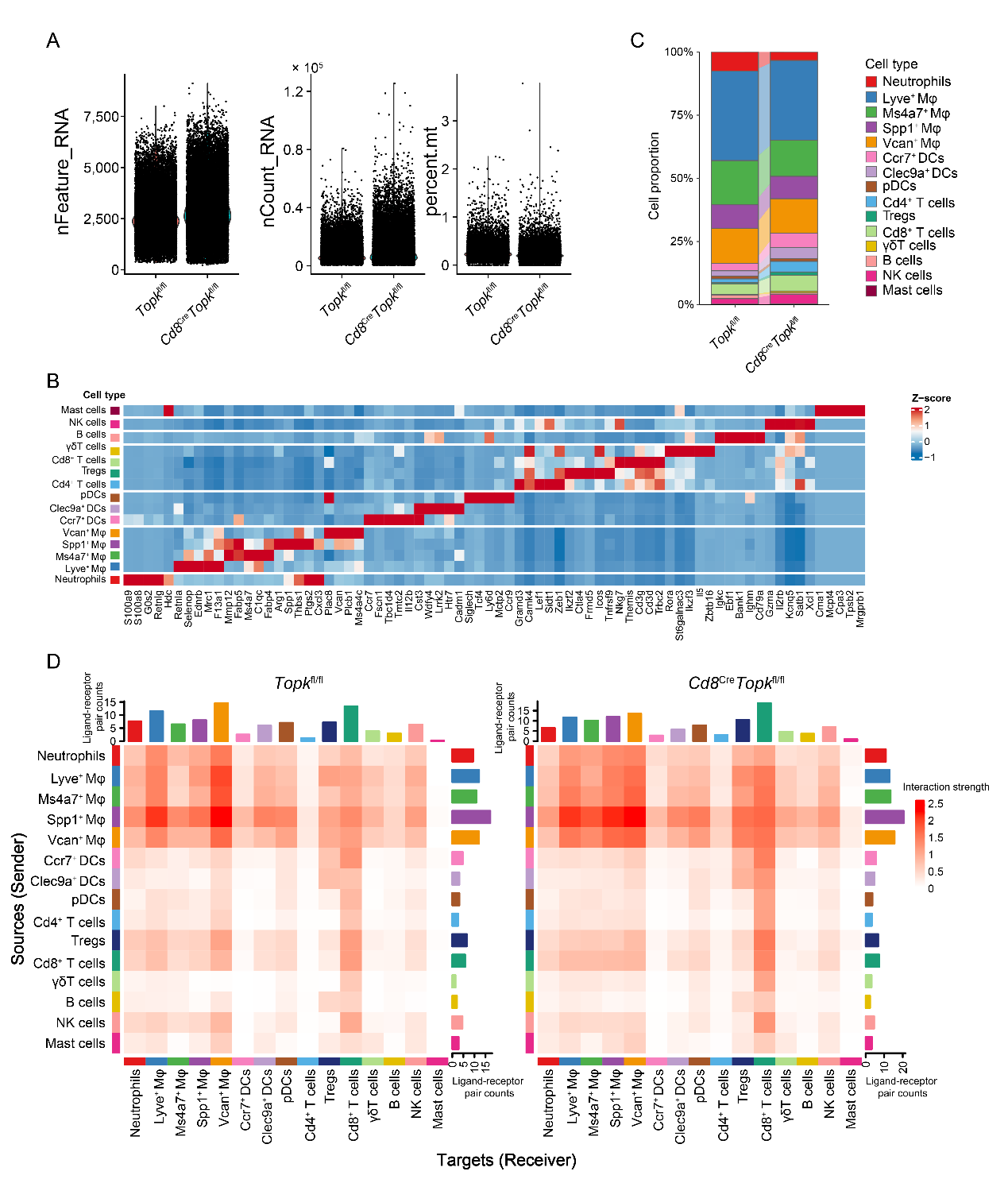


**Supplementary Figure S10.** **scRNA-seq quality control and supplementary analysis.**

Single-cell compositional analysis of TILs in tumor tissues derived from *Topk*^fl/fl^ and *Cd8^Cre^Topk*^fl/fl^ mice. Each group comprised 3 mice.

(A) scRNA-seq quality metrics including nFeature_RNA, nCount_RNA and percent.mt (mitochondrial gene content).

(B) Canonical marker gene expression defining immune cell identities in Mast cells, NK cells, B cells, γδT cells, Cd8^+^ T cells, Tregs, Cd4^+^ T cells, pDCs, Clec9a^+^ DCs, Ccr7^+^ DCs, Vcan^+^ Mφ, Spp1^+^ Mφ, Ms4a7^+^ Mφ, Lyve^+^ Mφ, neutrophils.

(C) Stacked bar plots of neutrophils, Lyve^+^ Mφ, Ms4a7^+^ Mφ, Spp1^+^ Mφ, Vcan^+^ Mφ, Ccr7^+^ DCs, Clec9a^+^ DCs, pDCs, Cd4^+^ T cells, Tregs, Cd8^+^ T cells, γδT cells, B cells, NK cells, Mast cells in *Topk*^fl/fl^ and *Cd8*^Cre^*Topk*^fl/fl^ mice.

(D) Heatmap of cell-cell communication interaction strength (rows: sender; columns: receiver) inferred from CellChatDB.mouse ligand-receptor pairs. Bar plots summarize the total number of sender and receiver interactions (ligand-receptor pair counts) for each cell group.

Abbreviations: *Topk*^fl/fl^, *Topk* floxed mouse; *Cd8*^Cre^*Topk*^fl/fl^, CD8-specific *Topk* conditional knockout mouse; TIL, tumor-infiltrating lymphocyte; t-SNE, t-distributed stochastic neighbor embedding; neutrophil, neutrophil granulocyte; Mφ, macrophage; DC, dendritic cell; Treg, regulatory T cell; γδT cell, gamma delta T cell; NK cell, natural killer cell.


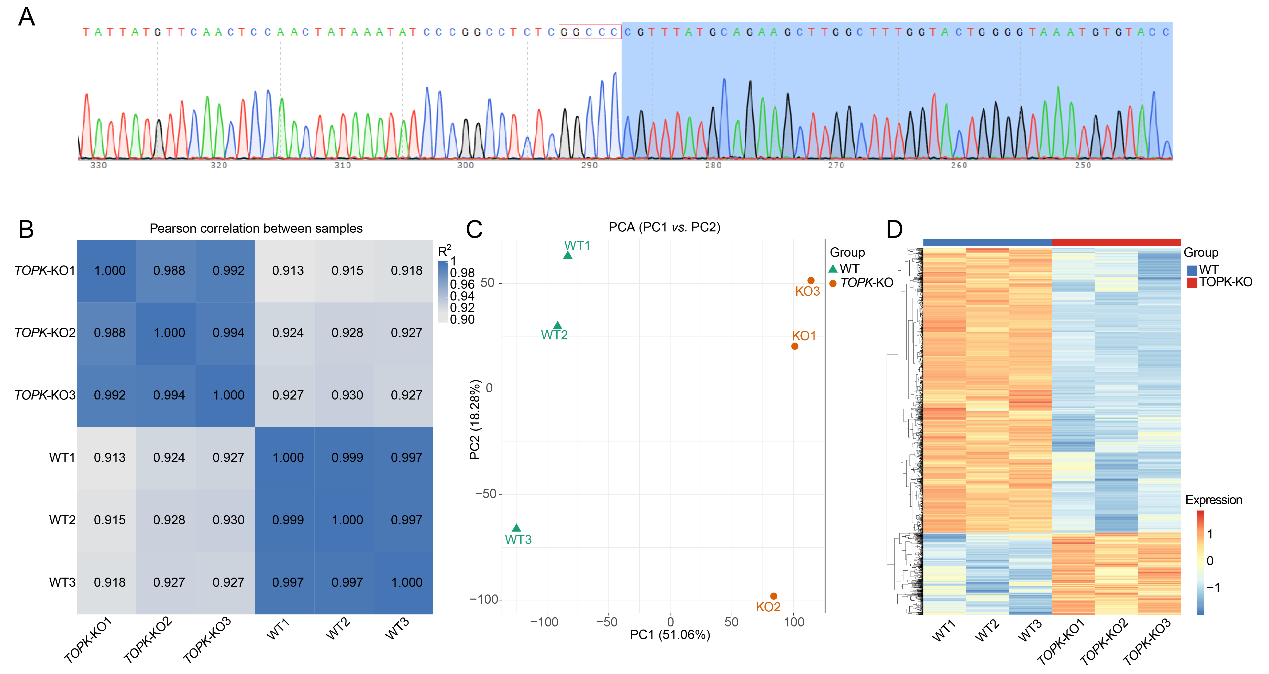


**Supplementary Figure S11.** **CRNA-seq quality assessment of WT and TOPK-KO Jurkat cells.**

RNA-seq analysis was performed on WT and TOPK-KO Jurkat cells. The data represent three independent experimental runs.

(A) CRISPR-Cas9-mediated TOPK-KO in Jurkat cells: a 5-bp insertion (GGCCC) was introduced into exon 3 of *TOPK*, inducing a frameshift mutation and premature termination.

(B-D) Transcriptomic profiling of WT and TOPK-KO Jurkat cells: inter-sample Pearson correlation matrix (B); PCA plot (PC1 *vs.* PC2, which capture the largest and second-largest sources of variance in gene expression, respectively) (C); heatmap of differentially expressed genes (FDR < 0.05) (D).

Abbreviations: RNA-seq, RNA sequencing; KO, knockout; WT, wild type; CRISPR, CRISPR-Cas9, clustered regularly interspaced short palindromic repeats-associated protein 9; PCA, principal component analysis.


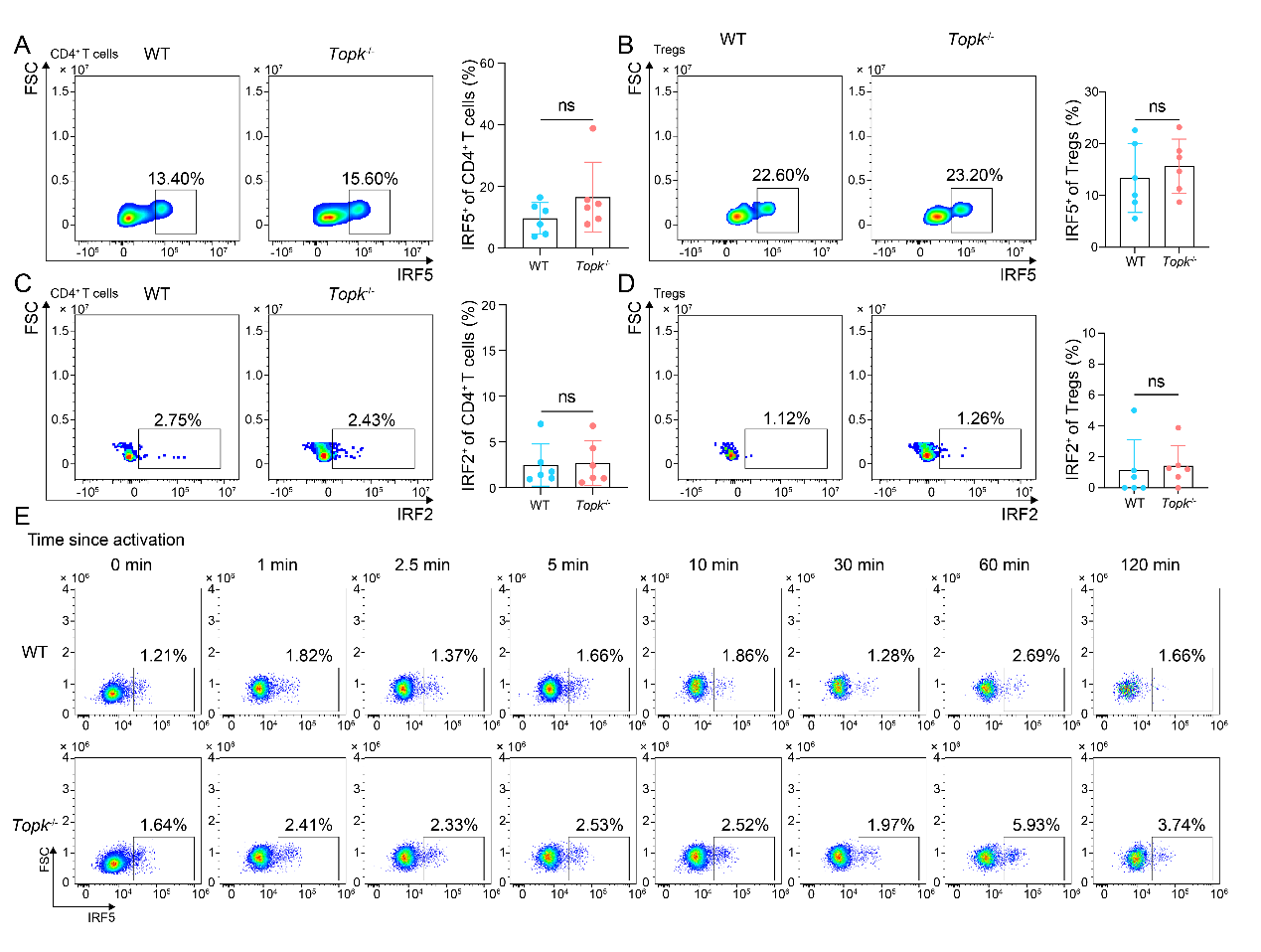


**Supplementary Figure S12. Analysis of IRF5 regulation by TOPK deficiency in T cell subsets.**

(A-D) *Topk*^-/-^ and WT mice were subcutaneously inoculated with 0.5 × 10⁶ B16-F10 cells, respectively (*n* = 6 per group). Data shown represent one of three independent experiments. Representative flow cytometry plots (left) and quantitative analysis (right) of the frequency of IRF5⁺ cells in CD4⁺ T cells (A) and Tregs (B) and IRF2⁺ cells in CD4⁺ T cells (C) and Tregs (D) from *Topk*^-/-^ and WT mice.

(E) WT and *Topk*^-/-^ CD8⁺ T cells) were stimulated with anti-CD3/CD28 Dynabeads (*n* = 6 mice per group). Divergence in IRF5^+^ cell proportions during *in vitro* activation of WT and *Topk*^-/-^ CD8^+^ T cells.

Data are shown as the mean ± SEM (A-D). Unpaired two-tailed *t*-tests (A-D). ns, not significant.

Abbreviations: WT, wild type; *Topk*^-/-^, *Topk* global knockout; Treg, regulatory T cell; IRF, interferon regulatory factor; SEM, standard error of the mean; min, minute.


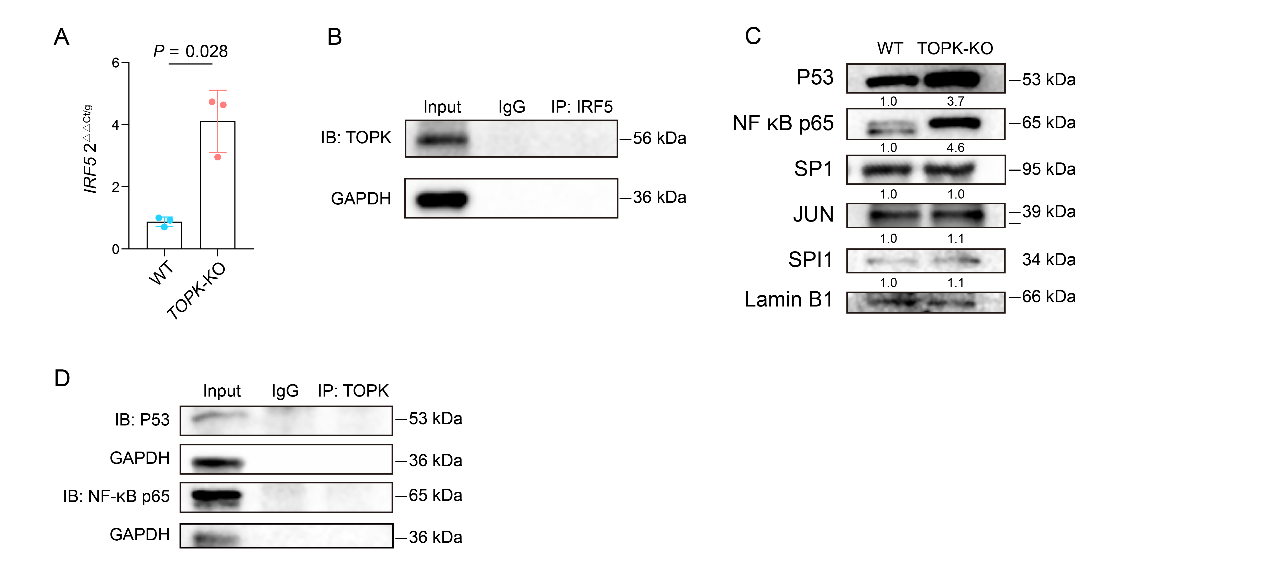


**Supplementary Figure S13.** **Mechanistic investigation of TOPK-mediated regulation of IRF5 expression in Jurkat cells.**

(A) Comparison of IRF5 mRNA levels between WT and TOPK-KO Jurkat cells.

(B) Co-IP analysis of the interaction between TOPK and IRF5.

(C) Western blotting analysis of the p53, NF-κB p65, SP1, JUN, SPI1 in WT and TOPK-KO Jurkat cells.

(D) Co-IP analysis of interactions between TOPK and the indicated transcription factors (D).

Data are shown as the mean ± SEM, unpaired two-tailed *t*-tests (A).

Abbreviations: WT, wild type; KO, knockout; co-IP, co-immunoprecipitation; SEM, standard error of the mean; TOPK, T-LAK cell-originated protein kinase; GAPDH, glyceraldehyde-3-phosphate dehydrogenase; p53, tumor protein p53; NF-κB p65, nuclear factor kappa B subunit 3; SP1, Sp1 transcription factor; JUN, Jun proto-oncogene, AP-1 transcription factor subunit; SPI1, Spi-1 proto-oncogene.


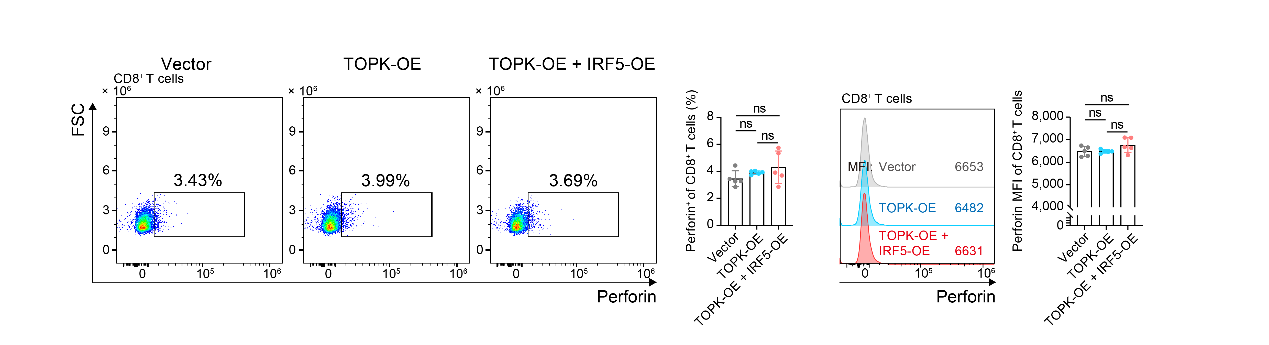


**Supplementary Figure S14. Analysis of cytokine of CD8^+^ T cells in TOPK and IRF5 rescue experiments.**

CD8⁺ T cells isolated from healthy donor PBMCs were activated using anti-CD3/CD28 Dynabeads and transduced with vector (control), TOPK-OE, or TOPK-OE + IRF5-OE, and co-cultured with A375 melanoma cells at an effector-to-target ratio of 10:1 for 48 h (*n* = 6 per group). Frequency (left) and MFI (right) of Perforin in CD8⁺ T cells across groups, shown as representative flow plots/histograms and quantification.

Representative flow cytometry analysis of CD8⁺ T cells for the positive rate and MFI of perforin across experiment groups. Data are shown as the mean ± SEM. Unpaired two-tailed *t*-tests. ns, not significant.

Abbreviations: PBMC, peripheral blood mononuclear cell; TOPK, T-LAK cell-originated protein kinase; IRF5, interferon regulatory factor 5; MFI, mean fluorescence intensity; SEM, standard error of the mean; OE, over expression.


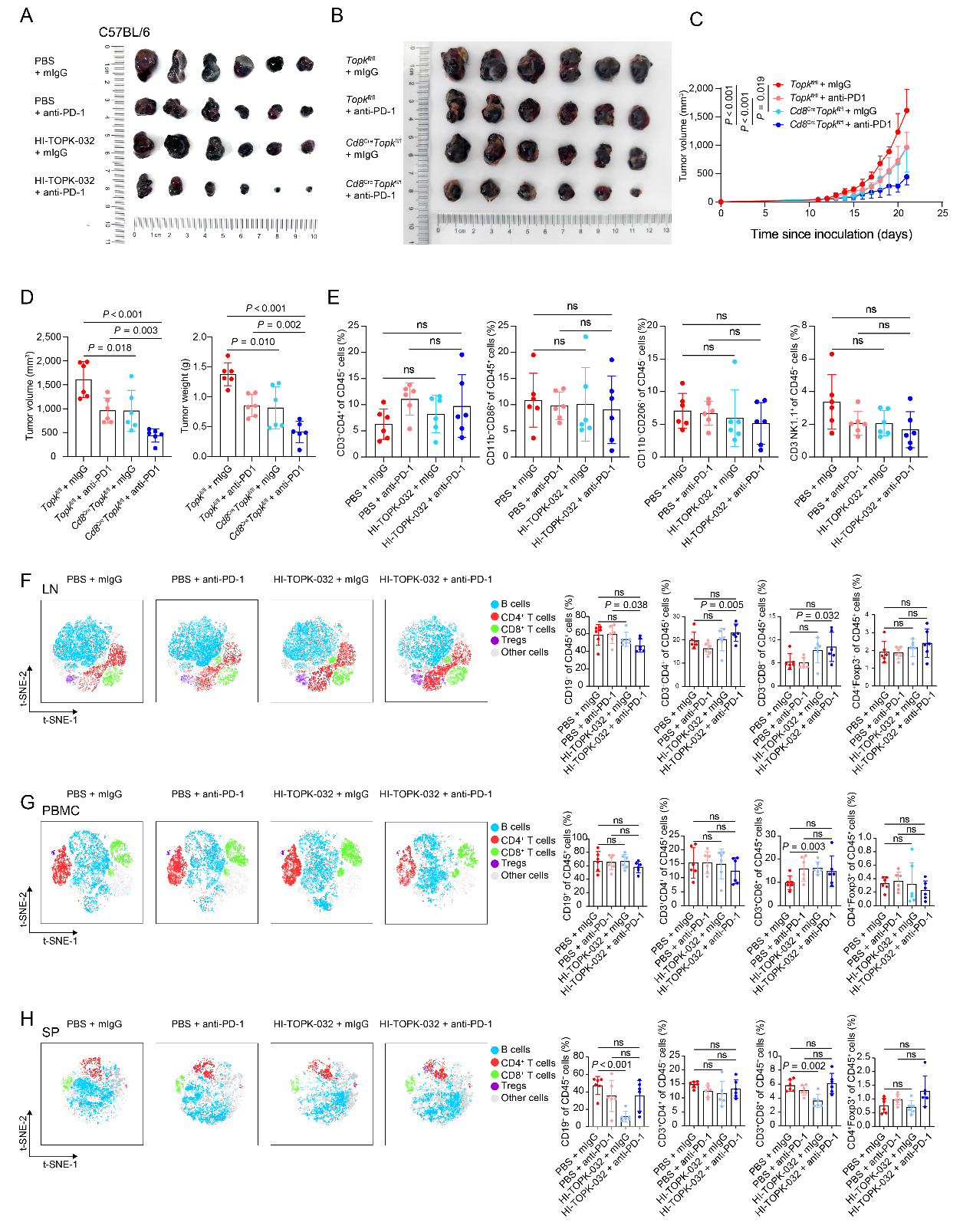


**Supplementary Figure S15. Analysis of immune cells dynamics in response to combined TOPK inhibition or deletion and anti-PD-1 therapy.**

(A) C57BL/6 mice bearing B16-F10 tumors (*n* = 6 per group) were treated with: PBS + mIgG; PBS + anti-PD-1; HI-TOPK-032 ​+ mIgG; HI-TOPK-032 + anti-PD-1. Image of C57BL/6 mice tumors in each treatment group.

(B-D) *Cd8*^Cre^*Topk*^fl/fl^ mice and *Topk*^fl/fl^ littermate controls (*n* = 6 per group) were inoculated subcutaneously with 0.5 × 10⁶ B16-F10 cells, and each genotype group was subsequently treated with anti-PD-1 or mIgG as an isotype control. Photographs of the tumor collected at day 21 since inoculation (B), tumor growth kinetics (C), tumor volumes (left) and weights (right) at day 21 since inoculation (D) in each group.

(E) Quantitative analysis of CD3^+^CD4^+^ T cells, CD11b^+^CD86^+^ M1, CD11b^+^CD206^+^ M2 and CD3^-^NK1.1^+^ NK cells of CD45^+^ TILs across experimental groups in A, based on flow cytometry data.

(F-H) t-SNE plots (left) and quantitative analysis (right) of immune cell subset proportion changes in the LNs (F), PBMCs (G), and SP (H) from C57BL/6 mice across the following treatment groups: PBS + mIgG, PBS + anti-PD-1, HI-TOPK-032 + mIgG, and HI-TOPK-032 + anti-PD-1 in the combined therapy.

Data are shown as the mean ± SEM. Mixed-effects model, group and time as fixed effects and individual mice as random effects (B), Unpaired two-tailed *t*-tests (D-H). ns, not significant.

Abbreviations: mIgG, mouse immunoglobulin G; HI-TOPK-032, TOPK-specific inhibitor; TIL, tumor-infiltrating lymphocyte; LN, lymph node; PBMC, peripheral blood mononuclear cell; SP, spleen; t-SNE, t-distributed stochastic neighbor embedding; SEM, standard error of the mean; Treg, regulatory T cell; M1/M2, macrophage type 1/2.


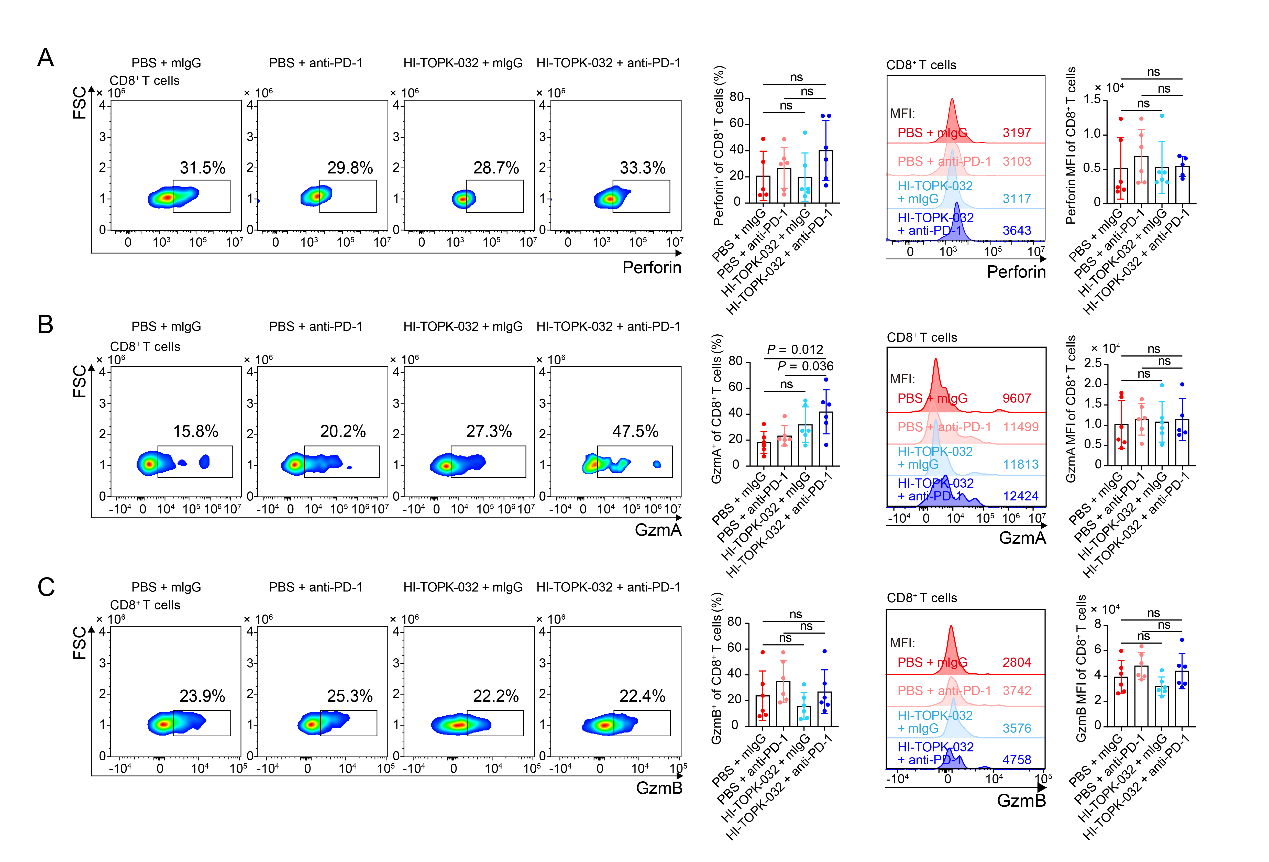


**Supplementary Figure S16. Analysis of cytotoxic profiles in response to combined HI-TOPK-032 and anti-PD-1 therapy.**

C57BL/6 mice bearing B16-F10 tumors (*n* = 6 per group) were treated with: PBS + mIgG; PBS + anti-PD-1; HI-TOPK-032​+ mIgG; HI-TOPK-032 + anti-PD-1.

Frequency (left) and MFI (right) of Perforin (A), GzmA (B) and GzmB (C) in CD8⁺ T cells across groups, shown as representative flow plots/histograms and quantification.

Data are shown as the mean ± SEM. Unpaired two-tailed *t*-tests. ns, not significant.

Abbreviations: mIgG, mouse immunoglobulin G; HI-TOPK-032, TOPK-specific inhibitor; TIL, tumor-infiltrating lymphocyte; GzmA/B, granzyme A/B; MFI, mean fluorescence intensity; SEM, standard error of the mean.
